# AD-LIBS: inferring ancestry across hybrid genomes using low-coverage sequence data

**DOI:** 10.1186/s12859-017-1613-0

**Published:** 2017-04-04

**Authors:** Nathan K. Schaefer, Beth Shapiro, Richard E. Green

**Affiliations:** 1 Department of Biomolecular Engineering, University of California, Santa Cruz, USA; 2 Department of Ecology and Evolutionary Biology, University of California, Santa Cruz, USA; 3 UCSC Genomics Institute, University of California, Santa Cruz, USA

**Keywords:** Hybridization, Admixture, Ancestry, Bears, Speciation

## Abstract

**Background:**

Inferring the ancestry of each region of admixed individuals’ genomes is useful in studies ranging from disease gene mapping to speciation genetics. Current methods require high-coverage genotype data and phased reference panels, and are therefore inappropriate for many data sets. We present a software application, AD-LIBS, that uses a hidden Markov model to infer ancestry across hybrid genomes without requiring variant calling or phasing. This approach is useful for non-model organisms and in cases of low-coverage data, such as ancient DNA.

**Results:**

We demonstrate the utility of AD-LIBS with synthetic data. We then use AD-LIBS to infer ancestry in two published data sets: European human genomes with Neanderthal ancestry and brown bear genomes with polar bear ancestry. AD-LIBS correctly infers 87–91% of ancestry in simulations and produces ancestry maps that agree with published results and global ancestry estimates in humans. In brown bears, we find more polar bear ancestry than has been published previously, using both AD-LIBS and an existing software application for local ancestry inference, HAPMIX. We validate AD-LIBS polar bear ancestry maps by recovering a geographic signal within bears that mirrors what is seen in SNP data. Finally, we demonstrate that AD-LIBS is more effective than HAPMIX at inferring ancestry when preexisting phased reference data are unavailable and genomes are sequenced to low coverage.

**Conclusions:**

AD-LIBS is an effective tool for ancestry inference that can be used even when few individuals are available for comparison or when genomes are sequenced to low coverage. AD-LIBS is therefore likely to be useful in studies of non-model or ancient organisms that lack large amounts of genomic DNA. AD-LIBS can therefore expand the range of studies in which admixture mapping is a viable tool.

**Electronic supplementary material:**

The online version of this article (doi:10.1186/s12859-017-1613-0) contains supplementary material, which is available to authorized users.

## Background

Inferring the ancestry of different parts of admixed diploid individuals’ genomes has been a goal of fields as diverse as disease gene mapping [1] and paleogenomics [2, 3]. Several computational approaches have been developed for ancestry detection. Among these, global methods calculate genome-wide amounts of admixture but do not attempt to localize admixture segments in the genome. In contrast, local methods for ancestry detection scan across admixed individuals’ genomes to search for haplotype blocks originating from specific ancestral populations [4]. Because haplotype blocks are broken down by recombination over time, local methods sacrifice power to detect very old admixture events in exchange for the ability to make specific, local statements about ancestry [4].

Many of the techniques for local ancestry inference were developed to investigate human ancestry and therefore incorporate assumptions that may not be valid for analyses of non-human data. For example, methods that compute on genotype calls rely on accurate calling. Genotype calling from sequence data as implemented in applications such as GATK [5] rely on pre-existing knowledge of polymorphic sites to make high-quality variant calls. This information is often unavailable for non-model organisms. In addition, some fields, such as paleogenomics [6], are limited by the amount of data that can possibly be recovered. Specifically, the degraded nature of recovered ancient DNA, and the upper limit imposed by the endogenous DNA content of source material [6], often results in coverage well below the threshold of 20X that has is considered necessary for reliable genotype calling [7]. Additionally, population genomic analyses may benefit more from sequencing many individuals to low coverage than sequencing fewer individuals more deeply [8], meaning that data collected for other types of analyses may not be suitable for ancestry inference techniques that rely on genotype calling. As an example, a recent study used ancient DNA from aurochs, the extinct wild ancestor of domestic cattle, to produce a ~6X coverage genome and infer gene flow into British and Irish cattle breeds post-domestication [9]. These data would be unsuited to current local ancestry inference techniques.

To address these challenges, we present AD-LIBS (Ancestry Detection through Length of Identity By State tracts), which is a software application that performs local ancestry inference by analyzing genetic data across genomic windows rather than at individual sites. AD-LIBS is designed for low-coverage shotgun resequencing data, and bypasses the need for variant calling and phasing. Input data for AD-LIBS is a single haploid sequence for each individual where every base is a random sample from one or the other chromosome, as has been done in other studies to mitigate genotyping errors [10, 11]. AD-LIBS uses a hidden Markov model to infer the most likely ancestral origin of each piece of the genome.

We test AD-LIBS using simulated data and find that it correctly infers 87–91% of ancestry, with a true positive rate of 89% for identifying admixed, homozygous regions and 82–85% for identifying admixed heterozygous regions.

We then use AD-LIBS to assign ancestry in two real data sets: one comprising five European humans with known Neanderthal ancestry and five West African individuals without Neanderthal ancestry, and another consisting of 18 brown bears from North America and Scandinavia with varying amounts of polar bear ancestry. In humans, we find that AD-LIBS produces maps of Neanderthal ancestry in Europeans that overlap significantly with published maps [2, 3] and global Neanderthal ancestry estimates that fall within 0–2% of what is expected from prior studies [12, 13]. In the bear data set, AD-LIBS identifies polar bear ancestry in all brown bear populations, including those believed previously to be unadmixed, and recovers a geographic signal in patterns of polar bear ancestry. We also test AD-LIBS on downsampled, artificially low-coverage data from bears and find that it produces consistent results down to about 2X genome-wide coverage, outperforming HAPMIX [14], an existing local ancestry inference tool, at coverage levels below this. In summary, AD-LIBS is an effective tool for producing local ancestry maps for genomes of hybrid individuals when only low-coverage sequence data are available and/or reference data are scarce.

## Results

### Overview of AD-LIBS

AD-LIBS (Ancestry Detection through Length of Identity-By-State tracts) uses a hidden Markov model to determine the ancestry of specific regions of hybrid individuals’ genomes inferred from low-coverage shotgun sequence data. To circumvent problems inherent in genotyping and phasing individuals sequenced to low coverage, AD-LIBS uses non-overlapping windows to scan pseudo-haploid sequence data, allowing all nucleotide positions in a given window to “vote” on the correct ancestry of that window. AD-LIBS does not require phased sequences from reference individuals nor does it require prior knowledge of polymorphic sites. AD-LIBS does require prior estimates of the population size, the number of generations since admixture, and the proportion of ancestry that the admixed population derives from each ancestral population. Although population size is best taken from census data, a rough estimate may be obtained from nucleotide diversity in the ancestral populations [15]. Admixture proportion may be estimated using the $\widehat{f}$ statistic [12, 16] if an outgroup genome is available, and time of admixture can be roughly inferred from the admixture proportion estimate together with prior knowledge about the ancestral species’ historical ranges and demography. AD-LIBS includes programs to calculate both average nucleotide diversity and $\widehat{f}$, and in practice, incorrect estimates for these parameters do not have a large effect on results (Additional file 1: Figure S1).

AD-LIBS scans across each hybrid individual’s genome in windows of a fixed width. In each window, AD-LIBS calculates a score based on average identity-by-state (IBS) tract lengths between the admixed individual and each individual from each ancestral population. AD-LIBS considers three possible types of ancestry in each window: homozygous for ancestry from one of the two ancestral populations, or heterozygous. Thus, AD-LIBS works as an ancestry genotyper for genomic segments and determines the most likely sequence of ancestry states across each chromosome or scaffold, given expected score distributions under each type of ancestry, computed from nucleotide diversity values. The probability of transitioning between ancestry states is related to the probability of recombination having occurred at specific genomic loci in the time since admixture, as well as the overall prevalence of alleles from each ancestral population in the admixed population.

AD-LIBS is designed to be efficient: its genome scanning and scoring components are written in C and its hidden Markov model component uses a Cython package (https://github.com/jmschrei/pomegranate). AD-LIBS requires that the system running it possesses enough memory to hold the longest chromosome or genomic scaffold sequence for each reference ancestral individual and a single hybrid in memory at once; for humans, this would comprise approximately 250 MB plus 250 MB RAM for each reference ancestral individual. On an Intel Xeon 2.7 GHz processor with 377 GB RAM, we ran AD-LIBS on a single 2.3 Gb hybrid brown bear genome, using ten ancestral reference genomes with numeric parameters pre-computed, in under 7.5 min. The same operation took approximately 9 min on a comparable machine with 70 GB RAM. When scanning multiple hybrid genomes, AD-LIBS can use multiple processes simultaneously to reduce execution time.

### Simulations

**Fig. 1:**
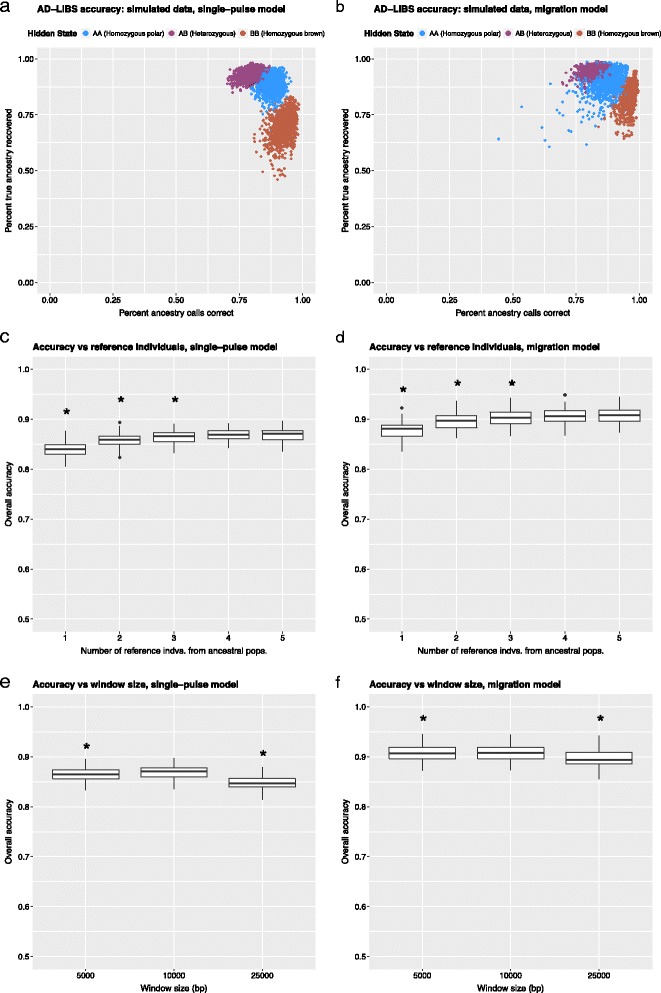
AD-LIBS accuracy using simulated data. **a**, **c**, and **e** refer to simulations with a single polar-brown bear admixture event 12,000 years ago, followed by isolation (single-pulse model); **b**, **d**, and **f** refer to simulations in which a brown-polar bear admixture event 12,000 years ago is followed by continual breeding with unadmixed brown bears (migration model). **a** and **b**: percent of AD-LIBS inferences correct and percent of true ancestry recovered in each ancestry state (homozygous polar bear, heterozygous, and homozygous brown bear) for each individual. **c** and **d**: Effect of using different numbers of reference ancestral individuals (1, 2, 3, 4, or 5 from each population) on overall accuracy, using 10kb windows. Asterisks denote a distribution mean significantly lower (*p* < 0.001) than the best distribution (5 individuals from both populations), according to *t*-test. **e** and **f**: Effect of using different window sizes (5kb, 10kb, and 25kb), with 5 reference bears from each ancestral population. Asterisks denote a distribution mean significantly lower (*p* < 0.001) than the best distribution (10kb windows), according to *t*-test

To assess the accuracy of AD-LIBS, we generated 100 simulated hybrid genomes, each consisting of ten, one-megabase (1 Mb) chromosomes. We assumed a demographic history resembling that of the ABC Islands brown bears, a well-studied population of brown bears known to have polar bear ancestry [10, 11, 17–19]. We used two demographic models, one with a single polar-brown bear admixture event 12,000 years ago (single-pulse model), and another incorporating continuous brown bear dispersal to the ABC Islands from the initial admixture event until the present (migration model) (see Implementation). We compared AD-LIBS ancestry calls to the known ancestry of each simulated chromosome. AD-LIBS performed well, with overall accuracy of 87% for the single-pulse model and 91% for the continuous migration model, and accurately recovered polar bear ancestry (82–85% true positive rate for heterozygous and 89% true positive rate for homozygous polar bear ancestry) (Table 1, Fig. 1). While choice of window size and number of reference individuals from each ancestral population had a small effect on overall accuracy, simulations show that suboptimal choices for both – e.g. one reference individual per ancestral population, or large windows of 25 kb – reduce overall accuracy only by several percent (Fig. 1). Additionally, we found that inaccurate prior estimates of admixed population size, number of generations since admixture, and polar bear ancestry proportion for individual hybrid bears had a similarly small effect on overall accuracy (Additional file 1: Figure S1).

**Table 1:** The accuracy of AD-LIBS ancestry inferences using simulated genomes

Model	Ancestry state	Prop. calls correct	Prop. truth detected
Migration	AA	0.8858	0.9310
Migration	AB	0.8447	0.9633
Migration	BB	0.9762	0.8459
Migration	Average	0.9022	0.9134
Migration	Overall accuracy	0.9069	
Single-pulse	AA	0.8933	0.9245
Single-pulse	AB	0.8170	0.9513
Single-pulse	BB	0.9501	0.7134
Single-pulse	Average	0.8868	0.8631
Single-pulse	Overall accuracy	0.8694	

Two demographic models representative of the ABC Islands bears’ history were used: one in which a single admixture event between polar and brown bears takes place 12,000 years ago, followed by isolation of the hybrid population (Single-pulse model), and one in which admixture takes place at the same time but is followed by continuous brown bear migration from the mainland (Migration model). Overall accuracy is the percent of all bases for which true ancestry matched AD-LIBS-inferred ancestry. Since this number is weighted toward more common ancestry states, the average across all three ancestry states is also given

**Fig. 2:**
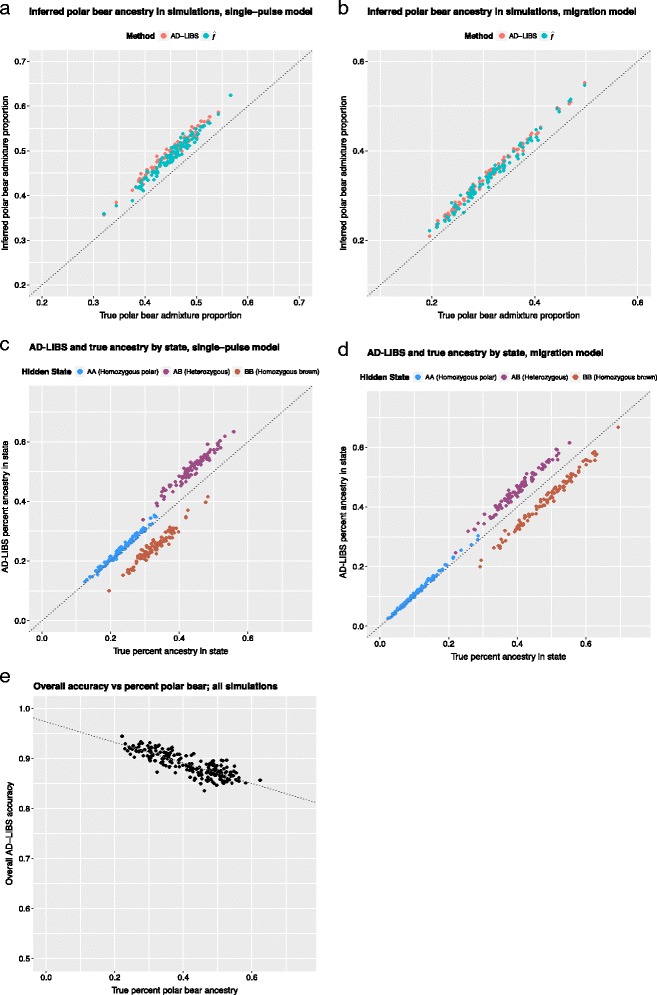
Accuracy of AD-LIBS estimates of the overall extent of polar bear ancestry, using simulated data. **a** and **c** refer to simulations with a single polar-brown bear admixture event 12,000 years ago, followed by isolation (single-pulse model); **b** and **d** refer to simulations in which a brown-polar bear admixture event 12,000 years ago is followed by continual breeding with unadmixed brown bears (migration model). All AD-LIBS runs in this figure used 5 reference individuals per ancestral population and 10kb windows. **a** and **b**: Inferred percent polar bear ancestry using AD-LIBS and $\widehat{f}$ versus true percent polar bear ancestry. **c** and **d**: inferred percent polar bear ancestry of each type, according to AD-LIBS, versus true percent polar bear ancestry of each type. Each point represents the percent of a single simulated hybrid bear genome with a specific type of ancestry. **e**: overall accuracy of AD-LIBS inferences versus true percent polar bear ancestry, including both types of simulations. The line of best fit by least-squares regression is also shown. Accuracy decreases slightly as polar bear ancestry increases, probably due to the tendency of AD-LIBS to overestimate the extent of heterozygous ancestry (**c** and **d**)

AD-LIBS was about as good at estimating each individual’s extent of polar bear ancestry as $\widehat{f}$, a widely-used statistic that estimates admixture proportion by comparing genome-wide frequencies of sites supporting tree topologies compatible and incompatible with admixture [12, 16]. AD-LIBS tends to overestimate the amount of heterozygous ancestry by several percent, however (Fig. 2). This might explain why AD-LIBS was more accurate in identifying ancestry under the migration model than the single-pulse model (Table 1, Fig. 1a, b). Genomes simulated under the migration model tend to have a lower overall extent of polar bear ancestry (Fig. 2a, b), giving AD-LIBS less opportunity to overestimate heterozygous ancestry. This causes the overall accuracy of AD-LIBS to fall by a rate of approximately 0.2% per percent polar bear ancestry (Fig. 2e; slope of best fit line by least squares regression = -0.206; adjusted *r* ^2^ = 0.687; F-statisic *p*-value < 2.2e–16, via linear model function in R [20]), although this effect may level off as higher levels of polar bear ancestry will lead to greater amounts of homozygous polar bear ancestry, which AD-LIBS detects more accurately.

### Real data

We collected two data sets for our study. First, we obtained five CEPH European (CEU) human genomes with Neanderthal ancestry [12] and five Yoruban (YRI) human genomes with little to no Neanderthal ancestry [12] from the 1000 Genomes Project [21], along with a single high-quality Neanderthal genome [13]. We used these data to map Neanderthal ancestry in Europeans using AD-LIBS and compare the results to previously-published local ancestry maps [2, 3] and global estimates of Neanderthal ancestry in Europeans [12, 13]. We also collected previously published shotgun sequence data from four polar bears, eighteen North American brown bears, and one American black bear [10, 11, 17, 18]. For a full list of bear samples used in this study, see Table 2. All reads were aligned to the polar bear reference genome [17] before pseudo-haploidization and variant calling (Implementation). The black bear was used as an outgroup to perform $\widehat{f}$ [12, 16] calculations for comparison with our findings.

**Table 2:** Sample details

Sample	Species	Location	Sex	Coverage	Study
PB7	*U. maritimus*	Spitsbergen, Svalbard	F	176.2X	Miller et al. 2012 [18]
PB12	*U. maritimus*	Qaanaq, Greenland	F	26.0X	Liu et al. 2014 [17]
PB68	*U. maritimus*	Qaanaq, Greenland	F	26.1X	Liu et al. 2014 [17]
PB105	*U. maritimus*	Disko West, Greenland	F	26.2X	Liu et al. 2014 [17]
OFS01	*U. arctos*	Östanvik, Sweden	F	22.8X	Liu et al. 2014 [17]
RF01	*U. arctos*	Ruokolahti, Finland	F	20.9X	Liu et al. 2014 [17]
SJS01	*U. arctos*	Slakka, Sweden	F	15.2X	Liu et al. 2014 [17]
Swe	*U. arctos*	Dalarna, Sweden	F	11.0X	Cahill et al. 2015 [11]
Den	*U. arctos*	Denali Natl. Park, AK	F	12.1X	Cahill et al. 2013 [10]
GP01	*U. arctos*	Glacier Park, Montana	M	16.8X	Liu et al. 2014 [17]
GRZ	*U. arctos*	Kenai Peninsula, AK	F	83.6X	Miller et al. 2012 [18]
ABC01	*U. arctos*	Baranof Island, AK	M	20.0X	Liu et al. 2014 [17]
ABC02	*U. arctos*	Baranof Island, AK	F	18.4X	Liu et al. 2014 [17]
ABC03	*U. arctos*	Chichagof Island, AK	M	19.6X	Liu et al. 2014 [17]
ABC04	*U. arctos*	Chichagof Island, AK	M	18.8X	Liu et al. 2014 [17]
ABC05	*U. arctos*	Chichagof Island, AK	F	22.4X	Liu et al. 2014 [17]
ABC06	*U. arctos*	Admiralty Island, AK	F	19.5X	Liu et al. 2014 [17]
Adm1	*U. arctos*	Admiralty Island, AK	F	12.1X	Cahill et al. 2013 [10]
Adm2	*U. arctos*	Admirality Island, AK	F	76.5X	Miller et al. 2012 [18]
Bar	*U. arctos*	Baranof Island, AK	M	49.1X	Miller et al. 2012 [18]
Chi1	*U. arctos*	Chichagof Island, AK	F	9.2X	Cahill et al. 2015 [11]
Chi2	*U. arctos*	Chichagof Island, AK	F	10.2X	Cahill et al. 2015 [11]
Uam	*U. americanus*	Pennsylvania	M	11.6X	Cahill et al. 2013 [10]

All sequence data were published in previous studies and downloaded as reads from the NCBI SRA. Coverage levels shown were estimated from numbers of raw reads before mapping to the reference genome. All samples were aligned to the polar bear reference genome, then subjected to base and map quality filtering, indel realignment, and duplicate removal

### Neanderthal ancestry in humans

AD-LIBS produced maps of Neanderthal ancestry in modern Europeans that agreed with published data, including global estimates of Neanderthal ancestry proportion [12, 13] as well as population-specific local ancestry maps [2, 3]. We prepared pseudo-haploid genome sequences from five randomly selected admixed CEPH European (CEU) and five unadmixed Yoruban (YRI) individuals from the 1000 Genomes Project [21], as well as two “haplotype” sequences from the Altai Neanderthal [13] (variants were randomly assigned to one or the other haplotype at heterozygous sites; see Implementation). We then ran AD-LIBS to infer Neanderthal ancestry in each European, using Neanderthal and Yoruban sequences as reference ancestral populations. For comparison, we also calculated each European individual’s Neanderthal ancestry via $\widehat{f}$. Although choice of window size affects results, the estimate of each individual’s Neanderthal ancestry proportion from AD-LIBS, using appropriate parameters, is 0.80–1.90% greater than the $\widehat{f}$ estimate (Table 3). AD-LIBS estimates are also 0.22–1.92% greater than the published $\widehat{f}$ estimate of 1.5–2.1% in all modern humans [13]. We note that back-migration of Europeans to West Africa has also contributed some Neanderthal ancestry to Yoruban individuals [13, 22], biasing $\widehat{f}$ estimates downward. We also compared the maps of Neanderthal ancestry from AD-LIBS to those published for CEPH Europeans [2, 3]. The AD-LIBS map overlapped significantly (p of greater overlap = 0 in 500 random trials) with the two previously published maps, although each map also finds Neanderthal ancestry in regions of the genome where the other maps do not.

**Table 3:** Results of running AD-LIBS on autosomal sequences of five randomly chosen European (CEU) individuals from the 1000 Genomes Project, with the Altai Neanderthal and five randomly chosen Yoruba (YRI) individuals from the 1000 Genomes Project as reference individuals from admixing populations

Individual	AD-LIBS, 10 kb	AD-LIBS, 15 kb	$\widehat{f}$
NA11832	11.5%	2.35%	1.41%
NA11840	11.5%	2.32%	1.52%
NA12340	13.8%	3.42%	1.52%
NA12383	13.4%	3.39%	1.56%
NA12814	13.6%	3.29%	1.45%

Using AD-LIBS with a window size (10 kb) lower than the recommended minimum of 14 kb gives bad results, while using a window size above this threshold (15 kb) gives much more reasonable results

Due to the nature of the emission probability distributions that AD-LIBS uses to distinguish between regions with different types of ancestry (Implementation), AD-LIBS produces inaccurate results when the window size is too small and/or too much genetic variation within the ancestral populations is also shared between them. This was the case when using 10 kb windows to scan for Neanderthal ancestry in Europeans (Table 3). When ancestral populations share a large amount of genetic variation, the distributions that AD-LIBS uses to distinguish between different types of ancestry tend to overlap. Using larger window sizes can reduce the variance of these distributions, and AD-LIBS can suggest an appropriate window size automatically (Implementation). Using a window size of 15 kb, above the threshold recommended by AD-LIBS, produced more accurate estimates of Neanderthal ancestry in Europeans (Table 3). When too much genetic variation within ancestral populations is also shared between them, however, AD-LIBS is unlikely to be accurate no matter what window size is chosen. This is likely to be the case when both admixing populations consist of modern humans, and this problem can be avoided by choosing an alternative to AD-LIBS when genetic differentiation between ancestral populations, as measured by statistics such as *F*_*ST*_ [23], is low.

### Polar bear ancestry in brown bears

We next used AD-LIBS to scan for polar bear ancestry in brown bears. We explored the effect of low sequence coverage depth on AD-LIBS inferences, compared global polar bear ancestry estimates from AD-LIBS to those produced using other techniques, and looked for geographic patterns in the distribution of polar bear ancestry across brown bear genomes.

#### Determining the necessary level of coverage

We sought to determine the effect of low sequence coverage depth on the accuracy of AD-LIBS by downsampling reads to produce artificial low-coverage genomes. We selected four admixed ABC Islands bears that were sequenced to at least 20X coverage (ABC01, ABC05, Adm2, and Bar), four polar bears over 20X coverage (PB7, PB12, PB68, and PB105) and three Scandinavian brown bears over 10X coverage (OFS01, RF01, and SJS01). The Scandinavian brown bears were hypothesized to be unadmixed with polar bears [17]. We obtained a set of variant calls and a pseudo-haploid genome sequence for each bear (Implementation), and downsampled every bear to 0.5X, 1X, 2X, 5X, and 10X coverage along the longest genomic scaffold (scaffold1) to produce a set of variant calls and a pseudo-haploid sequence for this scaffold at these different coverage levels. We ran AD-LIBS on each of the four admixed ABC Islands bears at full coverage and at each downsampled coverage level, using the three Scandinavian brown bears and four polar bears as unadmixed reference sequences. For comparison to AD-LIBS, we then ran HAPMIX [14], a commonly-used tool for local ancestry inference, on the same data. At each depth, we compared inferences from HAPMIX and AD-LIBS to the output of both programs run on the full-coverage data.

**Fig. 3:**
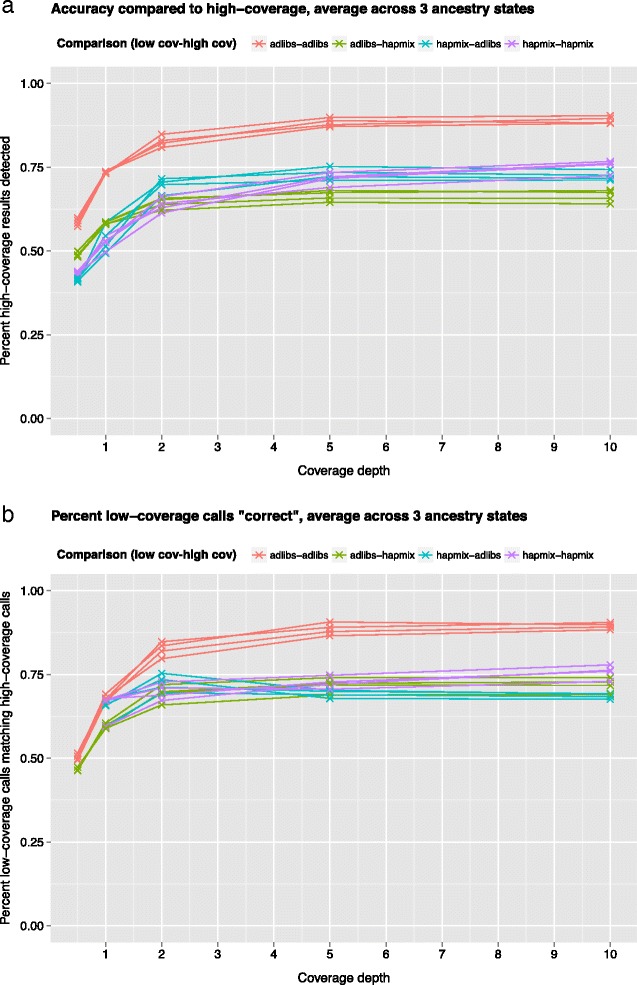
Results from downsampling four ABC Islands brown bears, three Scandinavian brown bears, and four polar bears to 0.5X, 1X, 2X, 5X, and 10X coverage along the longest genomic scaffold, running HAPMIX and AD-LIBS on the four ABC Islands bears at each coverage depth, and comparing these runs to results obtained from running both programs on the full-coverage versions of the same individuals. Each line represents an individual ABC Islands bear and each color represents a specific low coverage/full coverage comparison. **a**: percent of full coverage calls recovered by running each program at low coverage. Values given are averages across the three ancestry states (homozygous polar bear, heterozygous, and homozygous brown bear). **b**: percent of low coverage calls that were correct, according to full-coverage calls. Values given are averages across the three ancestry states. Some points are missing because HAPMIX was unable to detect any polar bear ancestry at 0.5X coverage

Using the high coverage data, we found that AD-LIBS and HAPMIX produce comparable results, although AD-LIBS tends to label regions heterozygous that HAPMIX labels homozygous polar bear (Table 4). At the lowest coverage levels, marker density after variant calling was too low for HAPMIX to produce interpretable results (Table 5). AD-LIBS more consistently infers the same ancestry at low and high coverage than does HAPMIX. Additionally, at coverage below 2X, ancestry calls made by AD-LIBS are more similar to the full-coverage ancestry calls from HAPMIX than are the low-coverage ancestry calls from HAPMIX (Fig. 3). When grouping results by ancestry state, low-coverage homozygous ancestry calls from AD-LIBS are more likely to match high-coverage calls than those from HAPMIX, although AD-LIBS labels some regions as heterozygous that are homozygous according to HAPMIX (Additional file 1: Figure S2). We infer from this experiment that AD-LIBS is consistent with itself down to about 2X coverage, and that inferences of homozygous ancestry from AD-LIBS are more reliable than those from HAPMIX at low coverage, although AD-LIBS erroneously labels some regions of homozygous ancestry heterozygous. By avoiding the need for variant calling, AD-LIBS also outperforms HAPMIX in cases of very low (0.5X or 1X) coverage, when there are not enough called variants to detect any polar bear ancestry. We note that genotype imputation could help improve marker density when running HAPMIX on low-coverage data, but this is only possible when studying species for which variant catalogs from large panels of reference individuals are available, such as humans.

**Table 4:** Percent ancestry of each type, as called by AD-LIBS and HAPMIX, in the four bears sequenced to sufficient coverage depth for variant calling

Bear	Ancestry state	AD-LIBS	HAPMIX	Agreement
ABC01	Hom. Polar	4.30%	7.30%	43.6%
ABC01	Heterozygous	28.7%	14.2%	46.7%
ABC01	Hom. Brown	67.1%	78.5%	84.2%
ABC01	Total	100%	100%	72.7%
ABC05	Hom. Polar	4.90%	8.39%	47.5%
ABC05	Heterozygous	28.3%	12.8%	44.2%
ABC05	Hom. Brown	66.8%	78.8%	84.0%
ABC05	Total	100%	100%	72.3%
Adm2	Hom. Polar	3.60%	6.52%	43.6%
Adm2	Heterozygous	26.8%	12.2%	42.6%
Adm2	Hom. Brown	69.6%	81.3%	84.7%
Adm2	Total	100%	100%	73.2%
Bar	Hom. Polar	4.56%	7.68%	46.6%
Bar	Heterozygous	27.8%	13.2%	44.6%
Bar	Hom. brown	67.6%	79.1%	84.3%
Bar	Total	100%	100%	72.8%

AD-LIBS calls more heterozygous ancestry than HAPMIX and probably overestimates heterozygous ancestry genome-wide

**Table 5:** Numbers of variants obtained from four polar bears (PB7, PB12, PB68, and PB105), three brown bears (OFS01, RF01, and SJS01), and four ABC Islands bears (ABC01, ABC05, Adm2, and Bar) at different levels of coverage along the longest polar bear genomic scaffold

Coverage level	# Raw variants	# Filtered	# Phased
10	490,705	410,174	410,174
5	443,470	288,761	288,821
2	353,433	44,706	44,706
1	268,595	1,494	1,494
0.5	172,214	14	14

At low coverage, marker density is too low for tools like HAPMIX to be useful or accurate

#### Measuring admixture proportion

We next sought to compare estimates of the genome-wide extent of polar bear ancestry in brown bears from AD-LIBS to those produced using other techniques. For each of eighteen brown bears, we ran AD-LIBS using four polar bears and four Scandinavian brown bears, the latter as potentially unadmixed models of ancestral populations (individual Scandinavian brown bears were excluded from the unadmixed reference brown bear set when treated as hybrid bears). For each bear, we also estimated genome-wide polar bear ancestry using the $\widehat{f}$ statistic [12, 16], which was used in prior studies of polar bear ancestry in brown bears [10, 11]. For this analysis, we used PB7 and PB12 as model polar bears, Swe as a model brown bear, and the American black bear as the outgroup. We also ran HAPMIX on all brown bears sequenced to at least 20X coverage, with all polar bears and the three Scandinavian brown bears above 20X coverage used as reference ancestral populations. For details about choices of parameters, see Implementation.

The admixture proportions detected with AD-LIBS were higher than our estimates using $\widehat{f}$ (Table 6). AD-LIBS-inferred admixture proportions are also higher than estimates from HAPMIX for the four ABC Islands bears of greater than 20X coverage (Table 6). We note that $\widehat{f}$ is considered a lower bound on admixture proportion, since it can only detect mutations that arose in the hybridizing lineages between the time of speciation and admixture [16]. This was not the case in our simulations, however, in which AD-LIBS and $\widehat{f}$ both consistently overestimated the polar bear admixture proportion by several percent (Fig. 2a, b). Although overestimation of heterozygous ancestry could explain why AD-LIBS produces erroneously high polar bear admixture proportions, an alternative explanation is needed to explain its discrepancy with $\widehat{f}$. One possibility is that in real data, selection in the brown bear lineage could reduce nucleotide diversity below the level typical of neutrally-evolving regions of the brown bear genome, but not below the level typical across the entire polar bear genome. This could cause windows of the genome in which brown bear-specific selection has taken place subsequent to the brown-polar bear split to appear erroneously heterozygous. It is also possible that polar bear ancestry in the Scandinavian brown bears, which were assumed to be unadmixed in $\widehat{f}$ calculations and in prior studies [10, 11], may also explain why $\widehat{f}$ estimates were lower than estimates using both AD-LIBS and HAPMIX.

**Table 6:** Percent polar bear for each brown bear in this study, calculated via $\widehat{f}$, AD-LIBS, and HAPMIX, if available

Bear	Origin	$\widehat{f}$	AD-LIBS	AD-LIBS conservative	HAPMIX
ABC01	Baranof Island, AK	8.63%	18.6%	15.5%	14.4%
ABC02	Baranof Island, AK	8.87%	18.8%	15.7%	14.8%
ABC03	Chichagof Island, AK	9.63%	19.4%	16.2%	N/A
ABC04	Chichagof Island, AK	9.03%	19.0%	15.8%	N/A
ABC05	Chichagof Island, AK	8.93%	19.1%	15.9%	N/A
ABC06	Admiralty Island, AK	6.56%	17.1%	14.2%	N/A
Adm1	Admiralty Island, AK	6.12%	16.6%	13.8%	N/A
Adm2	Admirality Island, AK	6.05%	17.0%	14.2%	12.6%
Bar	Baranof Island, AK	8.14%	18.5%	15.4%	14.3%
Chi1	Chichagof Island, AK	8.57%	18.6%	15.5%	N/A
Chi2	Chichagof Island, AK	8.69%	18.7%	15.6%	N/A
Den	Denali Natl. Park, AK	7.02%	14.5%	11.9%	N/A
GP01	Glacier Park, Montana	4.37%	17.2%	14.3%	N/A
GRZ	Kenai Peninsula, AK	3.30%	13.0%	10.7%	N/A
OFS01	Östanvik, Sweden	0.464%	5.35%	4.41%	N/A
RF01	Ruokolahti, Finland	0.319%	6.90%	5.67%	N/A
SJS01	Slakka, Sweden	0.211%	5.27%	4.33%	N/A
Swe	Dalarna, Sweden	0%*	4.89%	4.02%	N/A

The asterisk indicates that Swe was used as a model unadmixed brown bear in $\widehat{f}$ calculations, making polar bear ancestry undetectable. HAPMIX was only run on the four ABC Islands brown bears with minimum 20X coverage, to ensure that heterozygous variant calls were reliable. The “AD-LIBS conservative” column shows AD-LIBS estimates corrected according to the percent of homozygous and heterozygous polar bear ancestry calls that were incorrect in simulations under the single-pulse model (Table 1)

To be conservative, we recalculated all of our admixture proportions from AD-LIBS, this time multiplying the numbers of bases called homozygous and heterozygous polar bear by the rate at which these types of ancestry calls were correct in our simulations under the “single-pulse” model (Table 1) (Table 6, “AD-LIBS conservative” column). Since bases mis-called as heterozygous might actually be of either homozgyous polar bear or homozygous brown bear ancestry, treating them all as homozygous brown bear this way should produce an under-estimate of polar bear ancestry. The observation that AD-LIBS tends to find less homozygous polar bear ancestry than HAPMIX does (Table 4) also suggests that some of the mis-called heterozygous ancestry should be treated as homozygous polar bear ancestry. Regardless, we find using this technique that AD-LIBS still predicts more polar bear ancestry than $\widehat{f}$.

Comparing specific ancestry calls (homozygous polar bear, homozygous brown bear, and heterozygous) shows 72–74% overall agreement between AD-LIBS and HAPMIX, with most discrepancy resulting from AD-LIBS overestimating the extent of heterozygous ancestry (Tables 4 and 7). It is possible that HAPMIX underestimates homozygous polar bear ancestry as well, and that the problems described earlier with variant calling and phasing may lower the reliability of inferences from HAPMIX.

**Table 7:** Percent ancestry of each type called by AD-LIBS for all bears below 20X coverage, for which HAPMIX was not run

Bear	Hom, polar	Heterozygous	Hom. brown
ABC02	4.56%	28.5%	66.9%
ABC03	4.82%	29.1%	66.1%
ABC04	4.67%	28.6%	66.8%
ABC06	3.71%	26.7%	69.6%
Adm1	2.92%	27.4%	69.7%
Chi1	4.24%	28.7%	67.0%
Chi2	4.03%	29.3%	66.7%
Den	1.20%	26.6%	72.2%
GP01	3.43%	27.5%	69.1%
GRZ	1.72%	22.5%	75.8%
OFS01	0.441%	9.82%	89.7%
RF01	0.441%	12.9%	86.6%
SJS01	0.332%	9.88%	89.8%
Swe	0.339%	9.10%	90.6%

Heterozygous calls are probably overestimates

#### Shared patterns of ancestry

**Fig. 4:**
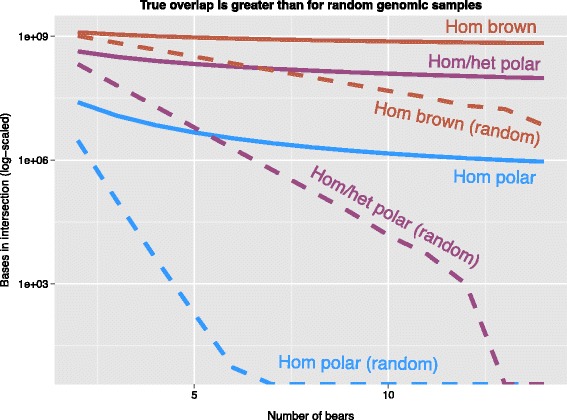
Comparing overlap of regions of different types of ancestry among hybrid bears. For every combination of 2 or more American brown bears, we measured the number of bases that AD-LIBS labeled with the same type of ancestry (homozygous polar, homozygous/heterozygous polar, or homozygous brown) in each bear. We also performed one random trial per real comparison, in which coordinates comprising random regions were sampled from the reference genome, producing sets of genome regions of the same size and number as the regions of ancestry produced by AD-LIBS for each bear, but randomly scattered across the genome. We then measured the overlap between these random ancestry regions for the sake of comparing to the true overlap. Averages of every comparison of each number of bears are shown as solid lines, and averages of every comparison of randomized versions of those same bears are shows as dashed lines

We next investigated the extent to which the same regions of the genome had the same type of ancestry in multiple bears. For each possible combination of two or more bears, we computed the number of bases in the genome for which all bears were inferred to have the same type of ancestry. Considering each type of ancestry separately, we then created random ancestry maps for each bear by sampling genomic coordinates comprising randomly-drawn regions of the same number and size from the reference genome. Computing the overlap among these random ancestry maps for all bears in the set gave us a null model against which to compare the extent of overlap among our true ancestry maps. For each group of bears and each type of ancestry, overlap is greater than for random samples (Fig. 4). This suggests that polar bear introgression took place within the shared demographic history of all of the brown bears in this study, as hypothesized by others [10, 11, 17–19].

**Fig. 5:**
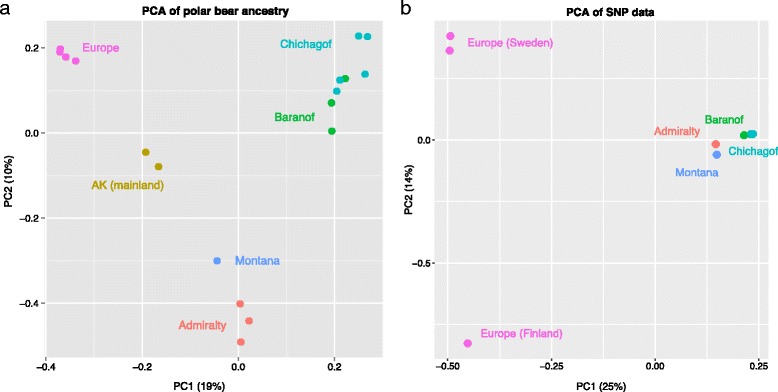
Geographic signal recovered in vectors of polar bear ancestry. **a**: principal components analysis (PCA) of polar bear ancestry state of 10kb genomic windows for 18 brown bears, using EIGENSOFT SmartPCA [24]. **b**: PCA of SNP data from a previous study [17], including a subset of the bears in **a**. Both plots show similar geographic patterns, with the Montana bear (GP01) falling close to the Admiralty Island bear(s), but only the SNP data separates Finnish (RF01) from Swedish brown bears

As another way to visualize sharing of polar bear-derived haplotypes among brown bears, we used principal components analysis (PCA), to test whether the ancestry data from AD-LIBS contain a similar geographic signal of admixture to that which has been observed previously from SNP data [17]. Using EIGENSOFT SmartPCA [24], we created vectors of ancestry across 10 kb genomic windows and performed PCA on these vectors for all 18 brown bears. Principal components place individuals into groups based on geography, with the first component corresponding to polar bear ancestry proportion. The ancestry results are largely similar to those from SNP data (Fig. 5). For example, the Montana bear clusters with the Admiralty Island individual(s), to the exclusion of the Baranof and Chichagof Island bears. The SNP PCA distinguishes bears from Finland and Sweden, however, while the ancestry PCA does not, suggesting that polar bear ancestry in these individuals might stem from the same historical event, despite different recent evolutionary histories.

## Discussion

AD-LIBS is a new technique for the detection and analysis of ancestry in admixed individuals, designed for use with low-coverage shotgun sequence data from non-model organisms. The technique works well on both simulated and real data, requires only several reference individuals from each ancestral population (Fig. 1), and is accurate at coverage depths as low as 2X (Fig. 3).

AD-LIBS is unlikely to perform as well as other local ancestry inference techniques when high-confidence genotype calls and phased data from reference populations are available. Moreover, AD-LIBS overestimates heterozygous ancestry (Fig. 2), although it has a lower false positive rate for identifying regions homozygous for one or the other type of ancestry (Fig. 1) and infers the correct amount of homozygous, introgressed ancestry genome-wide (Fig. 2). Therefore, one can be confident in results from AD-LIBS when analyzing genomic regions labeled as homozygous for ancestry from one or the other reference population but should use caution when describing regions heterozygous for ancestry.

Although AD-LIBS is robust to suboptimal choices of most parameters, window size must be chosen carefully, and *F*_*ST*_ between ancestral populations [23] must also be considered. The latter is important because overlap between the three emission probability distributions that AD-LIBS uses to determine which type of ancestry produced the set of IBS tract lengths in each window (see Implementation) depends to a large extent on *F*_*ST*_ between the two ancestral populations. If nucleotide diversity between populations is large relative to nucleotide diversity within populations, then the means of the emission probability distributions will lie further apart than distributions for ancestral populations with low *F*_*ST*_ (see Additional file 1: Supplementary Methods for expected emission probability distributions). While increasing the window size can help mitigate this problem by decreasing the variances of the distributions, it may be impossible to get accurate results when dealing with populations with low *F*_*ST*_ between them. As an example, AD-LIBS is not expected to give accurate results for human populations, in which within-population nucleotide diversity is often very similar to between-population nucleotide diversity. With populations of sufficiently high *F*_*ST*_, such as polar and brown bears, users should either allow AD-LIBS to determine an appropriate window size by measuring the overlap among emission probability distributions (see Implementation) or carefully evaluate results to ensure they are realistic. Using too small a window size to distinguish populations that are closely related can result in error (Table 3).

**Fig. 6:**
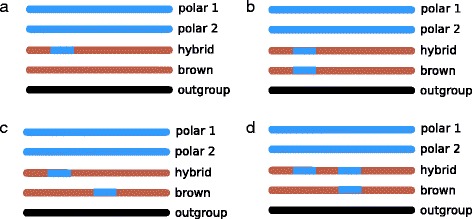
Illustration of cases where either a local ancestry detection method (like AD-LIBS) or a global ancestry detection (like $\widehat{f}$) might succeed, partially succeed, or fail. Each line represents a chromosome, with polar bear ancestry shown in blue and brown bear ancestry shown in brown. All five individuals needed for computation of $\widehat{f}$ are shown in each case. **a** local and global methods both succeed in detecting all of the hybrid individual’s polar bear ancestry. **b** local and global methods both fail to detect the hybrid individual’s polar bear ancestry. **c** local methods successfully detect the hybrid individual’s polar bear ancestry, since it is in a different part of the genome than the polar bear ancestry in the genome of the model “unadmixed” brown bear. Global methods fail to detect the hybrid individual’s polar bear ancestry. Since global methods use genome-wide averages, the hybrid individual is not seen to possess any more polar bear ancestry than the model “unadmixed” brown bear. **d** Both local and global methods will detect the hybrid’s first segment of polar bear ancestry but fail to detect the second segment, resulting in both types of methods underestimating the hybrid individual’s true percent polar bear ancestry

Using AD-LIBS, we detected a greater amount of polar bear ancestry in 18 brown bear genomes than has been previously reported using other methods [10, 11, 17, 18]. It is possible that these polar bear ancestry estimates are inflated by several percent due to AD-LIBS overestimating the extent of heterozygous ancestry (Fig. 2). If still valid, however, this finding illustrates an advantage of using local ancestry inference techniques like AD-LIBS over global techniques in admixture studies. If AD-LIBS is correctly inferring that Scandinavian brown bears have some polar bear ancestry, then prior studies that used these bears as model “unadmixed” brown bears may have underreported polar bear ancestry in all bears. The reason for this underreporting is that the global ancestry inference techniques used in prior studies, such as $\widehat{f}$, are genome-wide averages. As such, global ancestry inference methods cannot detect polar bear ancestry in an individual brown bear as long as the model “unadmixed” brown bear to which it is compared has the same amount of polar bear ancestry anywhere else in its genome. Local methods like AD-LIBS and HAPMIX, conversely, can detect polar bear ancestry at a particular genomic locus within an individual, as long as the model brown bear genome to which it is being compared is free of polar bear ancestry at that same locus (Fig. 6).

AD-LIBS maps of polar bear ancestry in brown bears also provide a look into the geographic history of polar-brown bear admixture. Given that principal components analysis (PCA) of polar bear ancestry groups bears geographically largely the same way as PCA of SNP data (Fig. 5), polar-brown bear admixture may have taken place before the present day North American brown bear populations formed. The placement of the Montana brown bear near bears from Admiralty Island in principal component (PC) space also suggests that ABC Islands brown bears could have been the source of polar bear ancestry in mainland brown bears, as previously hypothesized [11]. Finally, the existence of a small amount of polar bear ancestry in Scandinavian brown bears, which is similarly observed in Finnish and Swedish bears in PC space despite these bears’ clear difference in genotype PC space, suggests that there may have been a single, older polar-brown bear introgression event in Europe, independent from the source of polar bear ancestry in North American brown bears. If true, this result is evidence that hybridization between brown and polar bears may have been common in their evolutionary history, and may be the expected outcome of shifting habitat boundaries in times of global climate change.

## Conclusion

AD-LIBS expands the potential range of admixture analyses both to non-model organisms and to data sets in which only low-coverage genomes are available. While AD-LIBS should not replace existing approaches for high-coverage data or where phased reference panels are available, AD-LIBS accurately identifies genomic regions in hybrids that are homozygous for ancestry from a specific ancestral population, even with low coverage data. By thus reducing the quantity and quality of data needed, AD-LIBS can make admixture mapping a viable tool in a wider range of studies than was previously possible.

## Implementation

### Model description

AD-LIBS (Ancestry Detection through Length of Identity By State tracts) is designed for use with low-coverage sequence data from diploid organisms. The insight behind AD-LIBS is to consider windows of a genome, rather than individual SNP sites, when determining ancestry. This allows groups of variants to “vote” together on the ancestry of windows in the genome, decreasing the influence of individual sites that might be prone to genotyping or sequencing error. AD-LIBS takes as input pseudo-haploid FASTA sequences, in which every base is randomly sampled from one or the other homologous chromosome, rather than sets of genotype calls at variable sites. This eliminates the need for variant calling, which can be problematic without prior knowledge of polymorphic sites as in non-model organisms. It also avoids problems inherent in identifying heterozygous sites using low-coverage sequencing data [10, 11].

**Fig. 7:**
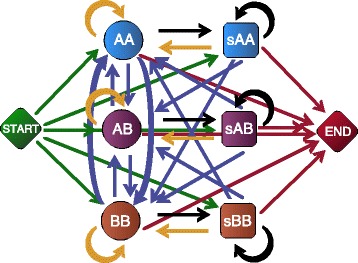
The state space of AD-LIBS hidden Markov model. The three round states (AA, AB, and BB) are ancestry states that can emit scores. AA represents regions where both homologous chromosomes derive ancestry from ancestral population A, AB represents regions of heterozygous ancestry, and BB represents regions homozygous for population B ancestry. The three square states (sAA, sAB, and sBB) are skip states, each associated with one of the three ancestry states. Skip states can only emit scores representing windows of the genome in which data are too sparse to infer ancestry. Each skip state is more likely to transition back to its associated ancestry state than to one of the others. Arrow colors represent different types of transition probabilities. Green arrows are starting probabilities and are related to the pre-estimated percent ancestry derived from each ancestral population (A and B). Blue arrows represent recombination events; their probability is related to the probability of a recombination event having happened at a given site in the time since admixture, as well as the probability of sampling a base from population A or B. Black arrows are related to the probability of skipping a given window, computed from the number of “N” bases encountered. Red arrows are transitions to the end state, with probabilities related to the number of windows on the chromosome or scaffold being scanned. Gold arrows represent probabilities that are computed after other probabilities, by subtracting from 1 the sum of all other transition probabilities out of a given state

If an individual has ancestors from both population A and B, each window of that individual’s genome can be classified as a sample of two chromosomes from population A, two from population B, or one of each. The state space of the hidden Markov Model (HMM) used by AD-LIBS therefore includes three ancestry states: AA, which models genomic windows in which both of an individual’s chromosomes descend from population A; AB, which models windows in which an individual derives one chromosome from each ancestral population; and BB, which models windows in which an individual is homozygous for ancestry from population B. Note that no attempt is made to “phase” variants when ancestry is heterozygous: AD-LIBS does not attempt to determine which of the two homologous chromosomes is of population A or B ancestry in the heterozygous state. In our model, we always designate the ancestral population with lower genetic diversity as population A and the other as population B. Figure 7 describes a cartoon of the HMM state space, including states not yet described. AD-LIBS uses the Python Pomegranate library for hidden Markov model operations, available at https://github.com/jmschrei/pomegranate.

### Transition probabilities

The transitions between states are related to the probability of recombination having occurred since admixture between the two ancestral populations. For this, AD-LIBS requires an estimate of the genome-wide extent of admixture and the number of generations since admixture. Given that the number of generations since admixture is *g*, and the per-nucleotide recombination probability per generation is *r*, the probability of a recombination event having taken place at a single nucleotide position in the time since admixture is $g r$. AD-LIBS assumes *r* to be a flat rate of 1 centimorgan per megabase, or 10^-8^ per site. If *p*, the extent of ancestry from population A in the admixed population, is known, then the probabilities of switching between state AA (homozygous population A ancestry), AB, (heterozygous ancestry), and BB (homozygous population B ancestry) can be determined. This requires considering the per-site probabilities of recombination events having happened or not in the time since admixture on both homologous chromosomes, along with the probabilities of the next base on each homologous chromosome being derived from population A or B. Additionally, AD-LIBS accounts for the effect of genetic drift: considering recombination events as alleles in the classic Wright-Fisher model, it derives the probability of resampling the same ancestral recombination event twice in a single individual, hereafter referred to as *z*. The probabilities of transitions between the three ancestry states are given in Table 8.

**Table 8:**
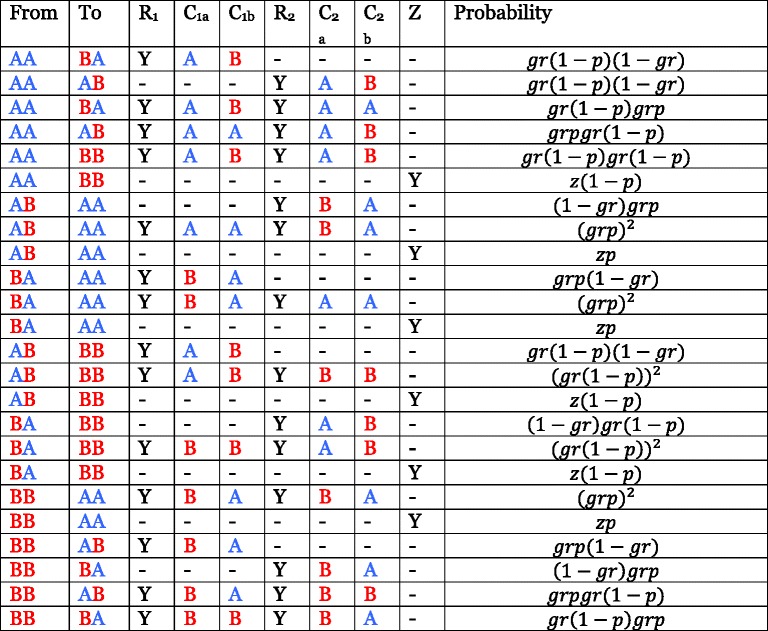
All possible combinations of events leading to transitions between the three ancestry states of the hidden Markov model

“A” and “B” denote chromosomes derived from ancestral populations A and B, and the three states AA, AB, and BB model regions where ancestry is homozygous from population A, heterozygous, and homozygous from population B, respectively (AB and BA are represented by same state, but are shown separately here to clarify that the ancestry of both separate chromosomes must be considered when computing probabilities). The other columns denote possible recombination-related events on the two parental homologues of a given chromosome (henceforth “homologue 1” and “homologue 2”). A “Y” in the R1 column signifies that recombination took place at a given base on chromosome homologue 1 in the time since admixture, and a “Y” in the R2 column signifies that recombination took place at this base on chromosome homologue 2. C1a and C1b refer to the ancestry of the bases on chromosome homologue 1 immediately before and after the recombination event, if it happened; C2a and C2b refer to the ancestry of the bases on chromosome homologue 2 before and after recombination. Z indicates that the same ancestral recombination event, which happened in the time since admixture, was sampled twice in the same individual (once on chromosome homologue 1 and once on chromosome homologue 2). The parameter g is the number of generations since admixture, r is the recombination probability per site per generation, assumed to be 1 cM/Mb or 10–8 per site, and z is the probability of resampling the same ancestral recombination event twice in one individual, according to genetic drift approximated by the Wright-Fisher model

As an example, two possible sets of events can lead to a transition from a region of homozygous population A ancestry (AA) to a region of homozygous population B ancestry (BB). One is that there has been a recombination event at the same site on both chromosome homologues in the time since admixture, the probability of which is ${\left( g r\right)}^2$, and that the base immediately after the recombination event is of population B ancestry on both chromosome homologues, the probability of which is (1 − *p*)^2^. This set of events thus has probability ${\left( g r\right)}^2{\left(1- p\right)}^2$. Conversely, if the two chromosome homologues have a recent common ancestor at the site of interest, it is possible that a historical recombination event between a region of population A ancestry and a region of population B ancestry happened once, but was inherited by both parents of the individual of interest. The probability of the individual inheriting the same historical recombination event from both parents is *z*, and the probability of the base immediately after this recombination event deriving from ancestral population B is (1 − *p*), making the probability of this set of events *z*(1 − *p*) (see Table 8).

Whereas *r* is a hard-coded approximation and *g* is a model parameter inferred from prior knowledge, the parameters *p* and *z* can be calculated. A popular method for estimating the admixture proportion *p* from sequence data is the statistic $\widehat{f}$, an extension of the D statistic used to estimate the extent of Neanderthal ancestry in modern humans [12, 25]. D is a genome-wide measure of excess derived allele sharing between an admixed individual and candidate introgressor; it compares numbers of sites, genome wide, that support alternative tree topologies. The statistic $\widehat{f}$ is a ratio of D computed on an admixed individual to D computed on an individual from the admixing population of interest. $\widehat{f}$ can be used to obtain a lower bound on admixture proportion. When *p* is not supplied by the user, AD-LIBS requires a genome from an outgroup individual and at least two individuals from admixing population A; these are used to compute $\widehat{f}$ as an approximation of *p*. Sometimes, for example when the test individual derives less of its genome from the introgressor than the individual hypothesized to be unadmixed, $\widehat{f}$ can yield negative values. In this case, and in every other case where *p* ≤ 0, we set *p* to a minimum value of 0.001. This allows AD-LIBS to detect regions of population A ancestry even when they were not originally expected, if the signal is strong enough.

The parameter *z*, or the probability of resampling the same ancestral recombination event twice in an individual, is less straightforward to compute. Conceptualizing recombination events of interest as alleles that arise within the admixed population during the time since admixture, with the per-site, per generation probability *r*, a Markov chain can be used to compute the probability of such a recombination event drifting to any frequency between 0 and $\frac{2 N}{2 N}$ where *N* is the number of individuals in the population, over the course of *g* generations [26]. This probability distribution can then be used to compute the probability of resampling the same recombination event twice in a single individual. Since the transition probability matrix for this Markov chain can become very large with large population sizes, making computation difficult, we implemented the solution to the diffusion approximation of this problem presented by McKane and Waxman [27] in AD-LIBS. For a detailed explanation of how the value of *z* is computed in AD-LIBS, see Additional file 1: Supplementary Methods.

In addition to the three ancestry states AA, AB, and BB, we defined three skip states, sAA, sAB, and sBB, which each model windows in which there is insufficient data to make an inference about ancestry (Fig. 7). Each of these states is only capable of emitting a designated score representative of low-quality windows. Each is also much more likely to transition back to its associated ancestry state than to one of the others: the transition probability *p*(AB|sAA) = *p*(AB|AA), *p*(BB|sAB) = *p*(BB|AB) and so on. This allows the HMM to have memory of the state in which a sequence was before encountering windows of sparse data: the probability of transitioning to a new ancestry state is the same, whether or not windows of sparse data are encountered. The probabilities of transitioning from ancestry states to skip states can only be calculated after scanning a sequence: windows with a percentage of ambiguous or “N” bases above a user-specified threshold are designated “skipped,” and the skip probability *s* is the number of skipped windows divided by the total number of windows in an input DNA sequence. The transition probability from each ancestry state to its associated skip state, as well as the probability of remaining in a skip state once there, is *s*. Since the emission probability distributions of skip states are very different from those of ancestry states, in practice the magnitude of *s* does not matter: windows intended to be skipped will be skipped whether *s* is high or low.

For a more detailed explanation of other transition probabilities, and how transition probabilities are set on sequences belonging to the X chromosome (or Z chromosome for species using the ZW sex determination system), see Additional file 1: Supplementary Methods.

### Emission probabilities

Rather than considering individual genotypes at known variable sites, AD-LIBS divides genomic sequences into windows and computes a score based on identity-by-state (IBS) tract lengths in each window. Identity-by-state tract lengths have proven useful in quantifying parameters of demography and admixture and underlie some popular methods for demographic inference [28, 29]. They are also easy to compute, can be measured without a set of high-confidence genotype calls, and have a clearly defined expected distribution, which should not be affected by the fact that our input data are pseudo-haploidized sequences rather than phased haplotypes.

AD-LIBS computes scores based on IBS tract lengths in fixed-width genomic windows. In each window, the “query” sequence from the hybrid individual is compared to all available sequences from ancestral populations A and B. The score *x* in a given window is $x= \log \left(\frac{1}{ a w}{\displaystyle {\sum}_{i=1}^a\left[\frac{1}{n}{\displaystyle {\sum}_{j=1}^n IB{S}_{i, j}}\right]}\right)-\kern0.75em  \log \left(\frac{1}{ b w}{\displaystyle {\sum}_{i=1}^b\left[\frac{1}{n}{\displaystyle {\sum}_{j=1}^n IB{S}_{i, j}}\right]}\right)$ where *a* is the number of sampled individuals from population A, *b* is the number of sampled individuals from population *b*, *n* is the total number of IBS tracts found in a given window between the hybrid and another individual, *IBS*_*i,j*_ is the length of the *j* ^th^ IBS tract with individual *i* sampled from either population A or B, and *w* is the window size in base pairs. In simpler terms, *x* is the ratio of the log transformed mean IBS tract length between the hybrid and individuals from population A, and between the hybrid and individuals from population B. For its emission probability distributions, AD-LIBS computes the expected distribution of *x* for each of the three ancestry states, with slight adjustments for scores in windows along the X (or Z) chromosome. For details, see Additional file 1: Supplementary Methods.

### Potential pitfalls

One parameter that must be chosen carefully is the window size. Apart from upper and lower bounds set on window size by mathematical limitations (see Additional file 1: Supplementary Methods), users have the ability to choose window sizes for their analyses. AD-LIBS can recommend a window size by testing the amount of overlap among the three emission probability distributions. Since overlap among emission probability distributions can hinder the ability of AD-LIBS to distinguish among different types of ancestry, and since the variance of all three distributions will decrease as window size increases (see Additional file 1: Supplementary Methods), increasing window size can improve discriminative power while risking failure to detect short ancestral haplotypes. AD-LIBS recommends a window size by computing the emission probability distributions for a range of window sizes beginning at the minimum bound. At each window size, it integrates a function returning the minimum value of each pair of distributions over those distributions’ full range, which gives a measure of overlap [30]. The smallest window size for which the maximum pairwise distribution overlap is 0.5 or lower is recommended. If the chosen window size causes the maximum overlap of any two of the three distributions to exceed 0.5, AD-LIBS iteratively multiplies the standard deviations of all three emission probability distributions by 0.5 and recomputes the overlap until it falls below 0.5. While this makes the model less realistic, it has the potential to improve discriminative power.

### Simulations

To test AD-LIBS, we used the December 11, 2009 version of Hudson’s coalescent simulator, ms [31], to simulate haplotypes under a demographic model representative of brown bears, polar bears, ABC Islands brown bears, and American black bears. We performed 20 trials in which ten 1 Mb pseudo-haploid chromosomes were generated for each of five polar bears, five mainland brown bears, five ABC Islands brown bears, and one black bear. Our demographic model is similar to that proposed by Cahill et al. [10], in which hybridization between brown and polar bears takes place on Alaska’s Admiralty, Baranof, and Chichagof (ABC) islands at the end of the Pleistocene epoch (the initial hybrid bear population consists of 50% polar bears and 50% brown bears). We chose for our model 0.94 Mya for the split time between the American black bear and brown and polar bears [32], 411 kya for the split between brown and polar bears [17], and 12 kya, the approximate end of the Pleistocene epoch, as the time of hybridization between mainland brown bears and the polar bears of the ABC islands [10]. Furthermore, we chose a generation time of 11.35 years and a per-site, per-generation mutation rate of 1.825728 ∗ 10^− 8^ [17], as well as a default recombination rate of 1 centimorgan per megabase, or 10^− 8^ per site. For nucleotide diversity values, we used *θ*_brown bear_ = 0.0017 and *θ*_polar bear_ = 4 ∗ 10^− 4^ estimated by Cahill et al. [10], along with *θ*_black bear_ = 0.0021 estimated by Kutschera et al. [32]; we converted these into effective population size values by dividing by four times the mutation rate. Our full ms command, which produced two haplotypes for each simulated individual, was ms 32 10 -t 1700.0 -r 931.135415571 1000000 -I 4 10 10 10 2 -n 1 0.235294117647 -n 4 1.23529411765 -es 0.0113546182949 2 0.5 -ej 0.0113546182949 2 1 -ej 0.0113546182949 5 3 -ej 0.3888956766 1 3 -ej 8.89445099767 3 4 –T.

In each of the 20 simulations, ms generated two black bear haplotypes and 10 each of polar bear, mainland brown bear, and ABC Islands brown bear haplotypes, with ten repetitions. After splitting the ms output files into individual repetitions, we then used Seq-Gen [33] with the Hasegawa, Kishino, and Yano (HKY) nucleotide substitution model [34] and a 4:1 transition:transversion ratio to convert each haplotype from each repetition into a DNA sequence. The full Seq-Gen command ms repetition output file was seq-gen -mHKY -t 4 -l 1000000 -s 0.0017 -p [number of trees in ms output file] [ms output file]. We then sampled two haplotypes per individual and, using a Python program, randomly choose the base from one or the other haplotype at each position to generate 1 Mb pseudo-haploid chromosome sequences. Finally, we concatenated the 1 Mb haploid sequences for each individual across the ten repetitions to yield 10 Mb simulated genomes for 5 polar bears, 5 mainland brown bears, 5 ABC Islands brown bears, and one black bear.

We used the trees from ms to produce maps of “true” ancestry for each hybrid bear in order to validate AD-LIBS results for each trial. We output trees with the –t parameter of ms and used these to produce BED files of the true ancestry of each segment of the simulated chromosomes for all five ABC Islands bears. To do this, we used a Python program to parse the trees describing the relationship of all simulated haplotypes at each segment of the simulated chromosome. For each ABC Islands brown bear haplotype at each segment, we computed the time to most recent common ancestor (TMRCA) with all polar bear haplotypes and with all brown bear haplotypes. In order to distinguish admixture from incomplete lineage sorting, we designated an ABC Islands bear haplotype as having polar bear ancestry only if its TMRCA with all polar bear haplotypes was more recent than its TMRCA with all brown bear haplotypes, and if its TMRCA with all polar bear haplotypes postdated the polar-brown bear split. If both haplotypes comprising a pseudo-haploid ABC Islands bear chromosome have polar bear ancestry in a given region, that region is designated “AA” for homozygous polar bear ancestry; if both have brown bear ancestry, it is designated “BB;” if there is one haplotype with each type of ancestry, it is designated “AB;” and if none of these is the case, no ancestry call is made. These are used as maps of “true” ancestry across the simulated chromosomes.

We then ran AD-LIBS on each simulated hybrid bear and assessed its accuracy using its map of “true” ancestry. For our initial estimate of polar bear ancestry in each hybrid bear, we calculated $\widehat{f}$ using the first two polar bear sequences, the first mainland brown bear sequence, and the black bear sequence as an outgroup. We then ran AD-LIBS with an admixed population size of 3000 and 1000 generations since admixture, and nucleotide diversity values that were calculated directly from the generated sequences. We note that only having 10 megabases of sequence for each bear may have hurt the accuracy of our $\widehat{f}$ calculations, since $\widehat{f}$ is a genome-wide average that requires a large number of single-site observations to disentangle true admixture from incomplete lineage sorting [12, 16]. For each simulated ABC Islands brown bear chromosome, we tried running AD-LIBS with one polar bear and one brown bear sequence to use as reference data, then two of each, three of each, four of each, and five of each, to determine whether the number of reference sequences affected output. We also used three different window sizes – 5 kb (slightly above the minimum threshold set by AD-LIBS, given the nucleotide diversity in the sequences), 10 kb, and 25 kb – for the same reason. After generating the results, we compared the output of AD-LIBS to the BED files of “true” ancestry by compiling the intersection of AD-LIBS’ ancestry features with the true ancestry features using BEDTools intersect [35] and determining the true ancestry across each window by majority vote of true ancestry regions contained within. For each state, then, we calculated the percent of bases for which the HMM’s classification was correct, as well as the percent of bases for which the true ancestry was recovered by the HMM. Admixture proportion was calculated as two times the number of bases in homozygous polar bear windows, plus the number of bases in heterozygous windows, all divided by two times the total number of windows for which AD-LIBS produced a label.

To compare performance, we repeated this experiment, but this time used a demographic model in which polar bears on the ABC islands are gradually converted into brown bears by continuous gene flow from mainland American brown bears. In this model, we allowed mainland bears to migrate to the ABC islands population at a rate of 0.001 (0.1% of each generation of the ABC Islands population is composed of brown bear migrants), beginning 12,000 years ago and continuing until the present. This model produces ABC Islands bears more varied in their polar bear ancestry proportion, and possibly more similar to the true ABC Islands bears. The full procedure for simulations with this model was the same as above, but using the ms command ms 32 10 -t 1700.0 -r 931.135415571 1000000 -I 4 10 10 10 2 -n 1 0.235294117647 -n 4 1.23529411765 -m 2 3 93.1135415571 -em 0.0113546182949 2 3 0 -ej 0.0113546182949 2 1 -ej 0.3888956766 1 3 -ej 8.89445099767 3 4 –T. We refer to the former model, with a single hybridization event, as the “single-pulse” model and the latter model, with continuous gene flow, as the “migration model.”

### Human and Neanderthal data

We ran AD-LIBS on human and Neanderthal data as a further test of AD-LIBS’s ability to correctly calculate admixture proportion, since high-coverage human and Neanderthal sequencing data are readily available, many studies have already sought to identify Neanderthal admixture proportions in modern humans and *F*_*ST*_ between humans and Neanderthals is reasonably high. We chose to scan European genomes for Neanderthal ancestry, because Neanderthal-human admixture is well studied, and we chose European over East Asian individuals because the history of Neanderthal-European gene flow may be simpler and involve fewer admixture events than that of Neanderthal-East Asian gene flow [3, 36, 37]. We randomly selected five European (CEU) individuals and five Yoruba (YRI) individuals from phase 3 of the 1000 Genomes Project [21], downloaded BAM files mapped to reference genome hg19 for each, and created a haploidized genomic sequence for each individual using the samtools mpileup utility [38] with map and base quality cutoffs of 20, along with a program that chooses a random base from the set that passed filters at every position, filtering out bases where coverage was greater than the 97.5^th^ percentile of coverage genome-wide. The European individuals used were NA11832, NA11840, NA12340, NA12383, and NA12814; the Yoruba were NA18504, NA18870, NA18934, NA19099, and NA19238. We then downloaded variant calls for the high-coverage Altai Neanderthal [13] and generated two “haplotype” sequences in hg19 coordinates using a program that transforms VCF to FASTA format, randomly assigning each variant at heterozygous sites to one or the other haplotype. Treating YRI and Altai as the two reference populations, we then calculated *π*_Altai_ = 0.000303, *π*_YRI_ = 0.001525, and *π*_Altai − YRI_ = 0.001763 from these sequences and chose a population size of 10,000, based on prior estimates [39], and 2,000 generations since admixture, roughly based on inferences drawn from Neanderthal haplotype block lengths in ancient human genomes [40, 41]. Neanderthal admixture proportions were estimated by calculating $\widehat{f}$ using both Neanderthal haplotype sequences, the Yoruba individual NA18504, and the reads from chimpanzee genome release PanTro4 [42], mapped to hg19 coordinates by the UCSC Genome Browser team [43]. After running AD-LIBS on each individual, we computed admixture proportion using the same technique as described in the Testing with simulated data section.

### Bear data preparation

Our bear sequence data were all published as part of previous studies [10, 11, 17, 18]; sample details are given in Table 2. We selected for study 11 hybrid brown/polar bears from Alaska’s Admiralty, Baranof, and Chichagof (ABC) islands (ABC01, ABC02, ABC03, ABC04, ABC05, ABC06, Adm1, Adm2, Bar, Chi1, and Chi2), one brown bear from Montana known to have polar bear ancestry (GP01), two brown bears with some polar bear ancestry from the Alaskan mainland (Den and GRZ), four Scandinavian brown bears hypothesized to be free of polar bear ancestry (OFS01, RF01, SJS01, and Swe), and four polar bears selected for high coverage depth (PB7, PB12, PB68, and PB105). Most data were downloaded as reads from the NCBI SRA, subjected to adapter removal and read merging using Seq-Prep (https://github.com/jstjohn/SeqPrep) and mapped to the polar bear reference genome [17] using BWA MEM [44, 45], sorted and indexed with samtools [38], and subjected to indel realignment via GATK, followed by duplicate removal via PicardTools [5]. The Denali park brown bear (Den), Swedish brown bear (Swe), Admiralty Island brown bear (Adm1), and American black bear (Uam), however, were processed as published in previous studies [10, 11]: adapter trimming using Trimmomatic [46], mapping using BWA aln [44], and duplicate removal using samtools rmdup [38] followed by GATK’s indel realignment [5]. Following this, we selected four polar bears (PB7, PB12, PB68, and PB105), two Scandinavian brown bears (OFS01 and RF01), and four ABC Islands brown bears (ABC01, ABC05, Adm2, and Bar), each of which had a minimum of 20X genome-wide average coverage, and performed variant calling on these using GATK’s Unified Genotyper. We set a minimum base and map quality of 30, and then discarded variants with a genotype quality lower than 30 or a variant quality lower than 50. We also filtered to exclude sites for which coverage was lower than 4 or greater than the 97.5^th^ genome-wide percentile for any individual bear; this yielded 16,635,425 SNPs and 3,054,975 indels. In addition to using these variant calls for downstream analysis, we used BEAGLE [47] with no reference panel, no imputation, and five iterations to phase our SNPs, resulting in a panel of 15,637,657 (94% of the original SNPs) phased polymorphic sites.

To compensate for our inability to reliably identify heterozygous sites in low-coverage (<20X) individuals, and to format our data for use with AD-LIBS, we generated pseudo-haploid sequences in reference genome coordinates for all bears by choosing a random base with minimum map and base quality of 30 at every site, skipping sites where coverage was greater than the 97.5^th^ percentile of genome-wide coverage [10, 11]. This was done using samtools mpileup with the polar bear reference genome and map and base quality filters, then piping to an in-house program that selects and outputs a random high-quality base at each position, yielding a FASTA file. Genome-wide coverage was computed using bedtools genomecov [35]. We then filtered these sequences to only scaffolds with a minimum length of 500 kb in the reference genome and calculated *π*_*polar*_ = 0.000615, *π*_*brown*_ = 0.00233, and *π*_*polar* − *brown*_ = 0.003564 using a utility included with AD-LIBS on these sequences. We then ran AD-LIBS on all bears, assuming an admixed population size of 3,000 and 2,000 generations since admixture, using PB105, PB12, OFS01, and Uam to estimate each admixed bear’s admixture proportion via $\widehat{f}$. For the Scandinavian bears OFS01, RF01, SJS01, and Swe, assumed to be free of polar bear admixture [11, 17], we specified an admixture proportion of 0.001 in order to allow the model to detect polar bear ancestry if it existed. We inferred ancestry for each of our brown bears using a window size of 10 kb (the size that worked best using simulations), a skip threshold of 0.25 (which gave very similar results to runs with skip thresholds of 0.1, 0.5, and 0.75), and using an X chromosome model for scaffolds determined belong to the X chromosome in a previous study [10]. We set the time since admixture to 1000 generations ago, the approximate end of the Pleistocene epoch assuming a generation time of 11.35 years [17] and an admixed population size of 3,000 individuals.

We also used our panel of phased SNPs to infer ancestry for our four ABC Islands bears that had at least a 20X average depth of coverage (ABC01, ABC05, Adm2, and Bar), using HAPMIX [14], with GENOTYPE = 1, OUTPUT_SITES = 1, THETA = 0.08, LAMBDA = 900.0, RECOMBINATION_VALS = 600 600, MUTATION_VALS = 0.2 0.2 0.01, and MISCOPYING_VALS = 0.05 0.05. This gave us an independent map of polar bear ancestry for these four bears against which to compare AD-LIBS’s results. We note that our HAPMIX results are not as reliable as those for human data, since our reference panel was phased computationally and thus subject to switch errors. After running HAPMIX, we converted output to BED files that could be compared to AD-LIBS results using an in-house program. This program assigns an ancestry state (homozygous population A, heterozygous, or homozygous population B) to each site by choosing the highest ancestry probability output by HAPMIX, or skipping sites where two or more probabilities are equal. It then merges runs of sites with the same ancestry into contiguous regions of ancestry and prints results in BED format.

### Low-coverage tests

To test AD-LIBS’s performance on low-coverage data, we used the same alignments as our full-coverage data. We chose to limit analysis, however, to the four hybrid ABC Islands bears for which we were able to run HAPMIX at full coverage (ABC01, ABC05, Adm2, and Bar), owing to the fact that these bears were sequenced to minimum 20X coverage and thus yielded reliable genotype calls. For unadmixed “reference” bears, we included all four polar bears (PB7, PB12, PB68, and PB105), as well as the three Scandinavian brown bears sequenced to at least 10X coverage (OFS01, RF01, and SJS01). We note that our full-coverage HAPMIX runs used only the Scandinavian bears over 20X coverage (OFS01 and RF01), and so our low-coverage HAPMIX runs actually had one more reference individual available than our high-coverage runs. For computational efficiency, we limited analysis to the longest scaffold of the polar bear reference genome (scaffold1, 67.4 Mb). For each bear, we compiled a random set of properly-paired reads that mapped to scaffold1 with minimum map quality 30 using samtools view, samtools bamshuf, and samtools bam2fq [38]. We then calculated, for each bear, the number of reads from these random sets required to obtain 0.5X, 1X, 2X, 5X, and 10X coverage across scaffold1. We then took subsets of our sets of high-quality mapping reads and, for each bear at each coverage level, mapped these reads to polar bear scaffold1 using BWA MEM [45], then performed GATK’s [5] indel realignment on the resulting BAM files. We did not remove duplicates, since the BAM files were already deduped prior to downsampling. We used our previously described strategy for preparing pseudo-haploid FASTA sequences (samtools mpileup and a program that randomly chooses a base at each position that passes quality filters), with map and base quality cutoffs of 20, to prepare data for use with AD-LIBS. We also used GATK’s UnifiedGenotyper to call SNPs along scaffold1 for every bear at each coverage level, removing sites with map or base quality below 20, as well as indel or non-biallelic variants. Following this, we phased variants using BEAGLE [47] with no reference panel, no imputation, and five iterations at each coverage level.

We ran HAPMIX on each bear for each coverage level using the same parameters as for full-coverage data (see Bear data preparation), and its results were converted to BED files for easy comparison to AD-LIBS’s results. AD-LIBS was then run on each hybrid bear at each coverage level with the same parameters as the full-genome runs, with the exception that nucleotide diversity values were computed from the haploidized FASTA files rather than using the previously-calculated values, the skip threshold was set to 0.75 to accommodate more missing data, and prior estimates of polar bear ancestry proportion were all set to 0.08, as they were in all HAPMIX runs. To compare output of HAPMIX and AD-LIBS runs to each other, we used the same technique as we did when comparing AD-LIBS results for simulated data to the BED files of true ancestry, described in the Simulations section.

### Shared polar bear ancestry

To test for sharing of the same types of ancestry across the same regions of the genomes in multiple bears, we used a custom Python program, along with several existing tools. We first created merged BED files of each specific type of ancestry for each bear using BEDTools [35], grouping heterozygous and homozygous ancestry together for one ancestry type. We also used BEDTools intersect to compute the size (in base pairs) of the intersection of each group of bears for each ancestry type, and random samples were taken from the polar bear genome using BEDTools shuffle, limited to the polar bear genomic scaffolds that were at least 500 kb long – the same set of scaffolds on which AD-LIBS was run.

In order to run EIGENSOFT SmartPCA [24] on the bear ancestry data, we used a custom script to convert AD-LIBS’s BED files into EIGENSTRAT format, using the starting coordinate of each window as the position of each “variant,” dropping “scaffold” from scaffold names, setting genetic distance to 0 (the default) for each “variant” so that a flat recombination rate can be assumed across each scaffold, and coding each homozygous polar bear window as 2, heterozygous windows as 1, and homozygous brown bear windows as 0.

For the SNP-based PCA run, we downloaded the set of polar and brown bear SNPs published as part of a recent study [17], excluded all polar bears, converted to EIGENSTRAT format, and ran EIGENSOFT SmartPCA [24] the same way as with our ancestry data. Only the first two principal components were considered.

## Additional file

Additional file 1: Contains Supplementary Methods. References for Supplementary Methods, **Figure S1** and **Figure S2.** (PDF 296 kb)

## Abbreviations

ABC: Admiralty, Baranof, and Chichagof (Islands)
GB: Gigabyte
HMM: Hidden Markov model
IBS: Identity-by-state
kb: Kilobase
kya: Thousand years ago
Mb: Megabase
MB: Megabyte
PCA: Principal components analysis
SNP: Single nucleotide polymorphism
TMRCA: Time to most recent common ancestor
