# Supplementary material for: AD-LIBS: inferring ancestry across hybrid genomes using low-coverage sequence data
Source: BMC Bioinformatics. 2017 Apr 4;18:203. doi: 10.1186/s12859-017-1613-0 (PMC5381037; doi:10.1186/s12859-017-1613-0)
Supplement: Additional file 1: — Contains Supplementary Methods. References for Supplementary Methods, Figure S1 and Figure S2. (PDF 296 kb) [file 12859_2017_1613_MOESM1_ESM.pdf]

Supplementary Methods

HMM transition probabilities: computing *z*

Transition probabilities in AD-LIBS depend primarily on the values of g, the number of generations since admixture, r, the recombination probability per site per generation, and z, the probability of resampling the same ancestral recombination event twice in a single individual (see Methods and Table 8). While g and r are simple to conceptualize and compute, z is more challenging, and we describe the computation of z in this section.

The Wright-Fisher model, in which populations are modeled as constant-sized unstructured groups of randomly mating individuals with non-overlapping generations, can be used to set up a calculation of z. Hartl and Clark [1] conceptualized allele frequencies under genetic drift in a Wright-Fisher population as a Markov chain problem: they described a state space of size 2N, where each state corresponds to an allele frequency in a diploid population of size N. Each entry in the transition probability matrix T, where T_{ij} is the probability of transitioning from i to j copies of an allele in a diploid population of size N in one generation, is given by $T_{ij} = \binom{2N}{j}\left(\frac{i}{2N}\right)^{j}\left(\frac{2N-i}{2N}\right)^{2N-j}$. Given that recombination events arise with probability r, then in this case $T_{0,j>0} = 2Nr^{j}$ and $T_{0,0} = 1 - \sum_{j=1}^{2N} T_{0,j}$ and the probability of a single recombination being resampled in an individual after allowing g generations of random drift is $\sum_{j=2}^{2N} T_{0,j}^{g}\left(\frac{j}{2N}\right)^{2}$. While conceptually appealing, this

formulation quickly becomes computationally intractable when there is a large population size N or number of generations since admixture g.

To improve computational efficiency, continuous approximations to the genetic drift problem, based on the mathematics of diffusion, have been proposed. Kimura [2] made an influential contribution, but his solution did not account for the so-called "absorbing" states of loss and fixation. We implemented the solution to the pure drift equation, without selection or migration, given by McKane and Waxman [3]. Given a population size N, a number of generations t, and an initial allele (or recombination) frequency of $1/2N$, the probability density of allele frequency x in the tth generation is given by $\sum_{n=0}^{\infty}\left[(2n+3)(n+1)(n+2)\ {}_2F_1(-n,n+3;2;\frac{1}{2N})\ {}_2F_1(-n,n+3;2;x)e^{-(n+1)(n+2)t/4N}\right]$, the probability of allele loss is a Dirac delta function with weight $\left(1-\frac{1}{2N}\right)\sum_{n=0}^{\infty}\left[\left(\frac{1}{2N}\right)(2n+3)\ {}_2F_1(-n,n+3;2;\frac{1}{2N})e^{-(n+1)(n+2)t/4N}\right]$, and the probability of allele fixation is a Dirac delta function with weight $\left(\frac{1}{2N}\right)\sum_{n=0}^{\infty}\left[\left(1-\frac{1}{2N}\right)(2n+3)(-1)^{n+1}\ {}_2F_1(-n,n+3;2;\frac{1}{2N})e^{-(n+1)(n+2)t/4N}\right]$, where ${}_2F_1(a,b;c;z)$ is the hypergeometric function.

In our implementation, we terminate the infinite sums when newly added terms are lower than 10^{-20} and we divide the allele frequency spectrum into 500 bins (or $2N$-1 bins, if $2N$-1 < 500), plus one bin each for the probabilities of allele loss and fixation. For any given number of generations t, we use the above equations to compute probability density at the

upper and lower limit of each bin, obtain probabilities from probability density via the trapezoid rule, and map the probability of each bin to the frequency value at its midpoint, giving a vector of probabilities V and a vector of allele frequencies F, each with 502 entries corresponding to bins indexed by b, where $b \sim [0, 501]$. We then iterate over each generation $0 \leq i < g$, and compute V and F for an allele that arose in generation i at frequency $\frac{1}{2N}$ and underwent $t = g - i$ generations of drift. For each of these vectors, then, we compute the per-site probability of resampling this allele (or recombination event) twice in an individual in generation g: if r is the recombination probability per site, $b \sim [0, 501]$ is the bin index, V_b is the probability of a given bin, and F_b is the mean allele frequency associated with that bin, then the probability of resampling the same allele or recombination event twice in an individual is $\sum_{b=0}^{501} r\, V_b {F_b}^2$. The overall probability of resampling the same ancestral recombination event twice in an individual is then $z = \sum_{i=0}^{g-1} \left[\sum_{b=0}^{501} r V_{i,b} {F_{i,b}}^2\right]$.

With these values, the transition probabilities between the three ancestry states can be computed per site as in Table 8. To transform these into transition probabilities between windows, each can be multiplied by the window size w, so that the transition probabilities between ancestry states are as follows:

$$p(\mathrm{AB} \mid \mathrm{AA}) = 2grw(1-p)(gpr - gr + 1)$$

$$p(\mathrm{BB} \mid \mathrm{AA}) = w(1-p)(-g^2pr^2 + g^2r^2 + z)$$

$$p(\mathrm{AA} \mid \mathrm{AB}) = 2pw(g^2pr^2 - g^2r^2 + gr + z)$$

$$p(\mathrm{BB} \mid \mathrm{AB}) = 2w(1-p)(-g^2pr^2 + gr + z)$$

$$p(\mathrm{AA} \mid \mathrm{BB}) = wp(g^2pr^2 + z)$$

$$p(\text{AB} \mid \text{BB}) = 2gprw(1 - gpr)$$

HMM transition probabilities

In addition to the three ancestry states and three skip states described in Methods, AD-LIBS also has start and end states. The probability of transitioning to the end state from any other state is $1/l$, where l is the number of windows in a genomic input sequence. The transition probabilities from the start state are based on the percent ancestry the admixed population derives from population A, p and the distribution of population A-like bases under Hardy-Weinberg equilibrium:

$$p(\text{AA} \mid \text{start}) = p^2(1 - s)$$

$$p(\text{sAA} \mid \text{start}) = p^2 s$$

$$p(\text{AB} \mid \text{start}) = 2p(1 - p)(1 - s)$$

$$p(\text{sAB} \mid \text{start}) = 2p(1 - p)s$$

$$p(\text{BB} \mid \text{start}) = (1 - p)^2(1 - s)$$

$$p(\text{sBB} \mid \text{start}) = (1 - p)^2 s$$

When all other probabilities have been determined, the probability of any ancestry state transitioning to itself is defined as one minus the sum of all other transition probabilities from that state. This is also how transition probabilities from skip states back to their associated ancestry states are determined. For example:

$$p(\text{AA} \mid \text{AA}) = 1 - p(\text{AB} \mid \text{AA}) - p(\text{BB} \mid \text{AA}) - p(\text{sAA} \mid \text{AA}) - p(\text{end} \mid \text{AA})$$

$$p(\text{AA} \mid \text{sAA}) = 1 - p(\text{sAA} \mid \text{sAA}) - p(\text{AB} \mid \text{sAA}) - p(\text{BB} \mid \text{sAA}) - p(\text{end} \mid \text{sAA})$$

AD-LIBS can also optionally account for the approximate general reduction in heterozygosity due to genetic drift, as formulated by Hartl and Clark [1]: given an initial

level of heterozygosity H_0, a population of N diploid individuals, the level of heterozygosity after t generations of genetic drift is $H_t \approx H_0 e^{-t/2N}$. This is approximated in AD-LIBS using $t =$ the number of generations since admixture g and population size N. We then reduce the probability of transitioning from any state to the heterozygous state according to this predicted reduction in heterozygosity, and compensate for this by increasing the probability of staying in or transitioning to one of the homozygous ancestry states:

$$p(\mathrm{AA} \mid \mathrm{AA}) = p(\mathrm{AA} \mid \mathrm{AA}) + \left(\frac{p(\mathrm{AA} \mid \mathrm{AA})}{p(\mathrm{AA} \mid \mathrm{AA}) + p(\mathrm{BB} \mid \mathrm{AA})}\right)\left(p(\mathrm{AB} \mid \mathrm{AA}) - p(\mathrm{AB} \mid \mathrm{AA}) * (H_t/H_0)\right)$$

$$p(\mathrm{AA} \mid \mathrm{sAA}) = p(\mathrm{AA} \mid \mathrm{sAA}) + \left(\frac{p(\mathrm{AA} \mid \mathrm{sAA})}{p(\mathrm{AA} \mid \mathrm{sAA}) + p(\mathrm{BB} \mid \mathrm{sAA})}\right)\left(p(\mathrm{AB} \mid \mathrm{sAA}) - p(\mathrm{AB} \mid \mathrm{sAA}) * (H_t/H_0)\right)$$

$$p(\mathrm{BB} \mid \mathrm{AA}) = p(\mathrm{BB} \mid \mathrm{AA}) + \left(\frac{p(\mathrm{BB} \mid \mathrm{AA})}{p(\mathrm{AA} \mid \mathrm{AA}) + p(\mathrm{BB} \mid \mathrm{AA})}\right)\left(p(\mathrm{AB} \mid \mathrm{AA}) - p(\mathrm{AB} \mid \mathrm{AA}) * (H_t/H_0)\right)$$

$$p(\mathrm{BB} \mid \mathrm{sAA}) = p(\mathrm{BB} \mid \mathrm{sAA}) + \left(\frac{p(\mathrm{BB} \mid \mathrm{sAA})}{p(\mathrm{AA} \mid \mathrm{sAA}) + p(\mathrm{BB} \mid \mathrm{sAA})}\right)\left(p(\mathrm{AB} \mid \mathrm{sAA}) - p(\mathrm{AB} \mid \mathrm{sAA}) * (H_t/H_0)\right)$$

$$p(\mathrm{AA} \mid \mathrm{BB}) = p(\mathrm{AA} \mid \mathrm{BB}) + \left(\frac{p(\mathrm{AA} \mid \mathrm{BB})}{p(\mathrm{AA} \mid \mathrm{BB}) + p(\mathrm{BB} \mid \mathrm{AA})}\right)\left(p(\mathrm{AB} \mid \mathrm{BB}) - p(\mathrm{AB} \mid \mathrm{BB}) * (H_t/H_0)\right)$$

$$p(\mathrm{AA} \mid \mathrm{sBB}) = p(\mathrm{AA} \mid \mathrm{sBB}) + \left(\frac{p(\mathrm{AA} \mid \mathrm{sBB})}{p(\mathrm{AA} \mid \mathrm{sBB}) + p(\mathrm{BB} \mid \mathrm{sAA})}\right)\left(p(\mathrm{AB} \mid \mathrm{sBB}) - p(\mathrm{AB} \mid \mathrm{sBB}) * (H_t/H_0)\right)$$

$$p(\mathrm{BB} \mid \mathrm{BB}) = p(\mathrm{BB} \mid \mathrm{BB}) + \left(\frac{p(\mathrm{BB} \mid \mathrm{BB})}{p(\mathrm{AA} \mid \mathrm{BB}) + p(\mathrm{BB} \mid \mathrm{BB})}\right)\left(p(\mathrm{AB} \mid \mathrm{BB}) - p(\mathrm{AB} \mid \mathrm{BB}) * (H_t/H_0)\right)$$

$$p(\mathrm{BB} \mid \mathrm{sBB}) = p(\mathrm{BB} \mid \mathrm{sBB}) + \left(\frac{p(\mathrm{BB} \mid \mathrm{sBB})}{p(\mathrm{AA} \mid \mathrm{sBB}) + p(\mathrm{BB} \mid \mathrm{sBB})}\right)\left(p(\mathrm{AB} \mid \mathrm{sBB}) - p(\mathrm{AB} \mid \mathrm{sBB}) * (H_t/H_0)\right)$$

$$p(\mathrm{AA} \mid \mathrm{AB}) = p(\mathrm{AA} \mid \mathrm{AB}) + \left(\frac{p(\mathrm{AA} \mid \mathrm{AB})}{p(\mathrm{AA} \mid \mathrm{AB}) + p(\mathrm{BB} \mid \mathrm{AB})}\right)\left(p(\mathrm{AB} \mid \mathrm{AB}) - p(\mathrm{AB} \mid \mathrm{AB}) * (H_t/H_0)\right)$$

$$p(\mathrm{AA} \mid \mathrm{sAB}) = p(\mathrm{AA} \mid \mathrm{sAB}) + \left(\frac{p(\mathrm{AA} \mid \mathrm{sAB})}{p(\mathrm{AA} \mid \mathrm{sAB}) + p(\mathrm{BB} \mid \mathrm{sAB})}\right)\left(p(\mathrm{AB} \mid \mathrm{sAB}) - p(\mathrm{AB} \mid \mathrm{sAB}) * (H_t/H_0)\right)$$

$$p(\mathrm{BB} \mid \mathrm{AB}) = p(\mathrm{BB} \mid \mathrm{AB}) + \left(\frac{p(\mathrm{BB} \mid \mathrm{AB})}{p(\mathrm{AA} \mid \mathrm{AB}) + p(\mathrm{BB} \mid \mathrm{AB})}\right)\left(p(\mathrm{AB} \mid \mathrm{AB}) - p(\mathrm{AB} \mid \mathrm{AB}) * (H_t/H_0)\right)$$

$$p(\mathrm{BB} \mid \mathrm{sAB}) = p(\mathrm{BB} \mid \mathrm{sAB}) + \left(\frac{p(\mathrm{BB} \mid \mathrm{sAB})}{p(\mathrm{AA} \mid \mathrm{sAB}) + p(\mathrm{BB} \mid \mathrm{sAB})}\right)\left(p(\mathrm{AB} \mid \mathrm{sAB}) - p(\mathrm{AB} \mid \mathrm{sAB}) * (H_t/H_0)\right)$$

$$p(\mathrm{AB} \mid \mathrm{AA}) = p(\mathrm{AB} \mid \mathrm{AA}) * (H_t/H_0)$$

$$p(\mathrm{AB} \mid \mathrm{sAA}) = p(\mathrm{AB} \mid \mathrm{sAA}) * (H_t/H_0)$$

$$p(\mathrm{AB} \mid \mathrm{AB}) = p(\mathrm{AB} \mid \mathrm{AB}) * (H_t/H_0)$$

$$p(\mathrm{AB} \mid \mathrm{sAB}) = p(\mathrm{AB} \mid \mathrm{sAB}) * (H_t/H_0)$$

$$p(\mathrm{AB} \mid \mathrm{BB}) = p(\mathrm{AB} \mid \mathrm{BB}) * (H_t/H_0)$$

$$p(\mathrm{AB} \mid \mathrm{sBB}) = p(\mathrm{AB} \mid \mathrm{sBB}) * (H_t/H_0)$$

Since many transition probabilities depend upon the window size w, we risk obtaining sums of transition probabilities from individual states that are greater than 1 when the window size is large. We take two steps to prevent this: first, we cap both the skip probability s and the probability of ending the sequence $1/l$ at maximum values of 0.05. In our experience, this number is high enough to still allow the model to detect skipped windows and to end sequences in the appropriate places. Second, given the other model parameters, we calculate the maximum possible window size that will allow the sum of transition probabilities out of each state to fall below 1. If the user chooses a window size that exceeds this threshold, the program notifies the user and exits.

Our implementation also considers some of the unique properties of the X chromosome [4]. Since there are fewer copies of the X chromosome than any autosome in a given population, the X chromosome only recombines approximately 2/3 as often as the autosomes. We therefore build a different model on chromosome sequences or genomic scaffolds specified to belong to the X chromosome: on these sequences, the r parameter is taken to be 2/3 of its default, autosomal value: $r_x = (2/3)r$. Furthermore, the z parameter quantifies genetic drift and thus depends on the population size N, but the effective population size of the X chromosome is 3/4 of the autosomal value, again owing to there being fewer copies of X chromosomes than autosomes in circulation in a population. We therefore recompute z using the same technique described earlier, but with $N_X = (3/4)N$, to give z_x, used in place of z on X chromosome sequences. Finally, users can specify which individuals are male, and for these individuals a haploid model is created instead of the default, diploid model on X chromosome sequences. In this model, there is no heterozygous ancestry state (AB) or

heterozygous skip state (sAB), and transitioning from one ancestry state to the other only requires a single recombination event, followed by sampling the next base from the set of bases of the opposite type of ancestry:

$$p(\text{BB} \mid \text{AA}) = gr(1-p)$$

$$p(\text{AA} \mid \text{BB}) = grp$$

The transition probabilities from the start state are also different in the haploid X chromosome model, due to the absence of the heterozygous state:

$$p(\text{AA} \mid \text{start}) = p(1-s)$$

$$p(\text{sAA} \mid \text{start}) = ps$$

$$p(\text{BB} \mid \text{start}) = (1-p)(1-s)$$

$$p(\text{sBB} \mid \text{start}) = (1-p)s$$

If the organisms under study follow the ZW rather than the XY sex determination system, the same concept holds, except that the “X chromosome sequences” supplied to AD-LIBS should be the names of sequences or genomic scaffolds belonging to the Z chromosome, and the “males” specified to AD-LIBS should actually be females.

Emission probability distributions: expectation

For a description of emission “scores” used by AD-LIBS, see Methods. In this section, we describe the expected distributions of these scores used by AD-LIBS.

The expected distribution of IBS tracts between two haplotypes depends only on the parameter π, or average nucleotide diversity per site between those two haplotypes. We therefore, as a first step, compute the average genome-wide nucleotide diversity per site

within population A, referred to as π_A, within population B, referred to as π_B, and between the two populations, referred to as π_{AB}. Given that π describes how often one expects to see a nucleotide difference, $1/\pi$ is the expected length of a haplotype before a difference is observed. IBS tract lengths tend to follow an exponential distribution with π as a parameter. Since our model considers samples of IBS tracts within genomic windows, however, we expect our mean IBS tract lengths to follow a normal distribution with a mean μ equal to the expected value and a standard deviation σ equal to the expected sample standard deviation, with the expected number of samples equal to the window size times π:

$$\mu = \frac{1}{w\pi}$$

$$\sigma = \frac{1}{\pi\sqrt{w\pi - 1}}$$

There are five such distributions to consider when computing scores. In genomic regions where a hybrid individual is homozygous for population A ancestry, sample mean IBS tracts with population A follow such a distribution with $\pi = \pi_A$ and sample mean IBS tracts with population B follow such a distribution with $\pi = \pi_{AB}$. Where there is homozygous population B ancestry, mean IBS lengths with population A have $\pi = \pi_{AB}$ and IBS lengths with population B have $\pi = \pi_B$. In heterozygous regions, mean IBS lengths with population A have $\pi = (\pi_A + \pi_{AB})/2$ and mean IBS lengths with population B have $\pi = (\pi_B + \pi_{AB})/2$. For each of these five distributions, we calculate normal μ and σ as above and then transform the standard deviation into the equivalent for a log-normal distribution:

$$\sigma' = \sqrt{\log\left(\frac{\sigma^2}{\mu^2} + 1\right)} = \sqrt{\log\left(\frac{w^2}{w\pi - 1} + 1\right)}$$

We then use these values to compute the parameters for the three emission probability distributions. Since each is the ratio of two distributions, the variances of the emission probability distributions are the sum of the variances of the two mean IBS tract length distributions they compare:

$$\sigma_{AA} = \sqrt{\log\left(\frac{w^2}{w\pi_A - 1} + 1\right) + \log\left(\frac{w^2}{w\pi_{AB} - 1} + 1\right)}$$

$$\sigma_{BB} = \sqrt{\log\left(\frac{w^2}{w\pi_{AB} - 1} + 1\right) + \log\left(\frac{w^2}{w\pi_B - 1} + 1\right)}$$

$$\sigma_{AB} = \sqrt{\log\left(\frac{w^2}{w\,(\pi_A + \pi_{AB})/2 - 1} + 1\right) + \log\left(\frac{w^2}{w\,(\pi_B + \pi_{AB})/2 - 1} + 1\right)}$$

The means of the three score distributions are the differences of the natural logarithms of the two sample mean IBS tract length distributions they compare:

$$\mu_{AA} = \log\left(\frac{1}{w\pi_A}\right) - \log\left(\frac{1}{w\pi_{AB}}\right)$$

$$\mu_{AB} = \log\left(\frac{1}{w\pi_{AB}}\right) - \log\left(\frac{1}{w\pi_B}\right)$$

$$\mu_{AB} = \log\left(\frac{1}{w\,(\pi_A + \pi_{AB})/2}\right) - \log\left(\frac{1}{w\,(\pi_B + \pi_{AB})/2}\right)$$

In AD-LIBS, all three emission probability distributions are modeled as normal distributions, due to successful performance on training data. We also reserve a specific value to be used as a "skip" score; distributions are set such that the skip score has zero probability under all three emission probability distributions. We also create an emission probability distribution for the three skip states that is only capable of emitting this value.

As with the transition probabilities, sequences belonging to the X chromosome present an edge case in which changes to the model are necessary. Since the effective population size of the X chromosome is 3/4 that of the autosomes, we multiply π_A, π_B, and π_{AB} by 3/4 before calculating the emission probability distribution parameters.

Whereas the need to keep transition probabilities below 1 sets an upper bound on window size, our expected emission probability distributions set a lower bound on window size. If π_A is the lowest value of π, then per the standard deviation calculations above, we require $w\pi_A > 1$ to avoid division by zero (standard deviation calculations can involve division by zero if the expected number of IBS tract observations in a given window is less than one). It is generally preferable to choose the smallest possible window size for which there is a reasonable lack of overlap among emission probability distributions (see Methods).

References for Supplementary Methods

1. Hartl DL, Clark AG: **Random Genetic Drift**. In *Principles of Population Genetics*. Fourth Edi. Sunderland, Massachussetts: Sinauer Associates, Inc.; 2007:102–118.

2. Kimura M: **Solution of a Process of Random Genetic Drift With a Continuous Model.** *Proc Natl Acad Sci U S A* 1955, **41**:144–150.

3. McKane a. J, Waxman D: **Singular solutions of the diffusion equation of population genetics**. *J Theor Biol* 2007, **247**:849–858.

4. Schaffner SF: **The X chromosome in population genetics**. *Nat Rev Genet* 2004, **5**(January):43–51.

Supplementary figures

Figure S 1

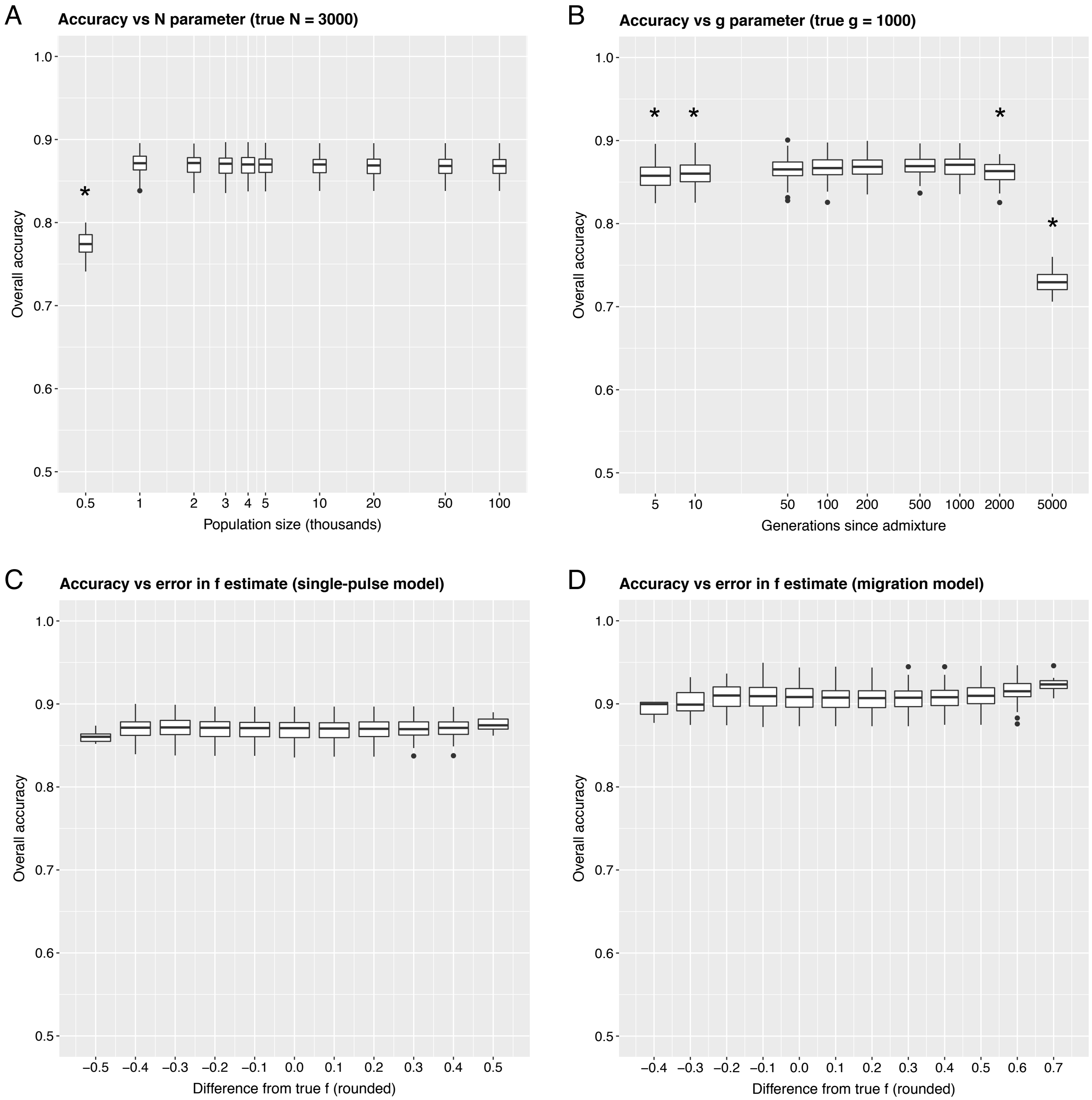

AD-LIBS accuracy on simulated data, using incorrect population parameters. Simulations here used the "single-pulse" admixture model (see Figure 1 A, C, and E) except where otherwise noted, with 10kb windows, which were automatically adjusted by AD-LIBS as necessary. Asterisks denote accuracy significantly lower ($p < 0.001$) than that obtained using correct parameters. A: AD-LIBS accuracy using different prior population size estimates (true N = 3000) and correct number of generations since admixture (1000). B: AD-LIBS accuracy using different estimates for the number of generations since admixture (true g = 1000) and correct population size (3000). C: AD-LIBS accuracy using prior estimates of polar bear admixture proportion that differed from the true value, rounded to the nearest 10%. D: Same as C, but using the "migration" admixture model (see Figure 1 B, D, and F), which produced a wider range of true admixture proportions.

Figure S 2

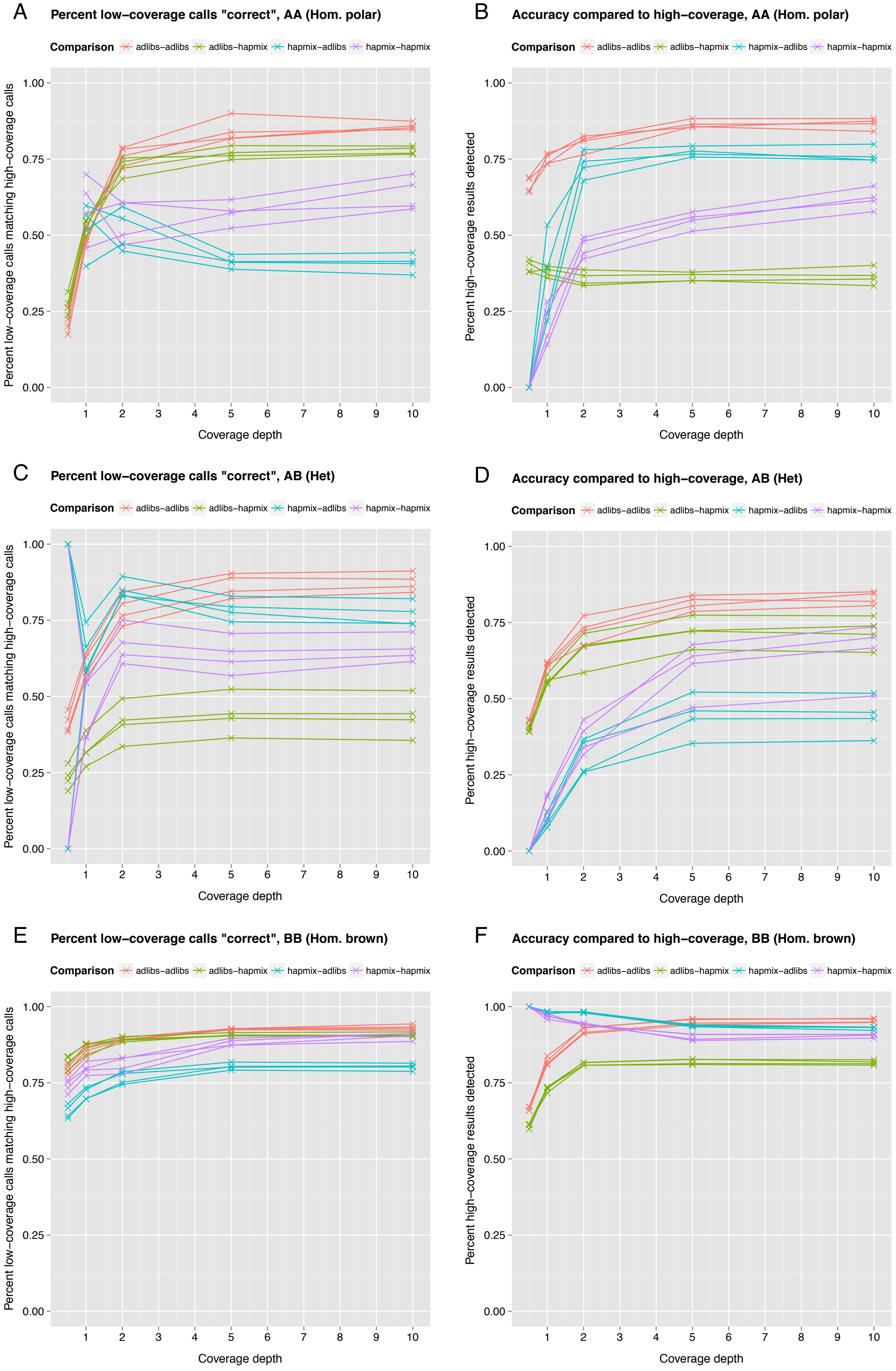

A
Percent low−coverage calls "correct", AA (Hom. polar)
Comparison adlibs−adlibs adlibs−hapmix hapmix−adlibs hapmix−hapmix
Percent low−coverage calls matching high−coverage calls
Coverage depth
B
Accuracy compared to high−coverage, AA (Hom. polar)
Comparison adlibs−adlibs adlibs−hapmix hapmix−adlibs hapmix−hapmix
Percent high−coverage results detected
Coverage depth
C
Percent low−coverage calls "correct", AB (Het)
Comparison adlibs−adlibs adlibs−hapmix hapmix−adlibs hapmix−hapmix
Percent low−coverage calls matching high−coverage calls
Coverage depth
D
Accuracy compared to high−coverage, AB (Het)
Comparison adlibs−adlibs adlibs−hapmix hapmix−adlibs hapmix−hapmix
Percent high−coverage results detected
Coverage depth
E
Percent low−coverage calls "correct", BB (Hom. brown)
Comparison adlibs−adlibs adlibs−hapmix hapmix−adlibs hapmix−hapmix
Percent low−coverage calls matching high−coverage calls
Coverage depth
F
Accuracy compared to high−coverage, BB (Hom. brown)
Comparison adlibs−adlibs adlibs−hapmix hapmix−adlibs hapmix−hapmix
Percent high−coverage results detected
Coverage depth

Results from downsampling four ABC Islands brown bears, three Scandinavian brown bears, and four polar bears to 0.5x, 1x, 2x, 5x, and 10x coverage along the longest genomic scaffold, running HAPMIX and AD-LIBS on the four ABC Islands bears at each coverage depth, and comparing these runs to results obtained from running both programs on the full-coverage versions of the same individuals. Each line represents an individual ABC Islands bear and each color represents a specific low coverage/full coverage comparison. A and B assess homozygous polar bear (AA) calls, C and D assess heterozygous (AB) calls, and E and F assess homozygous brown bear (BB) calls. A, C, and E measure the percent of low-coverage calls that were "correct" according to the high-coverage runs, while B, D, and F measure the percent of the high-coverage runs' calls that were also detected by the low-coverage runs. In almost every case, AD-LIBS is more consistent with itself than other comparisons. We also note that low-coverage AD-LIBS inferences of homozygous polar and brown bear ancestry are more often correct, according to HAPMIX run at full coverage, than HAPMIX run at low coverage (A and E). AD-LIBS may, however, erroneously call more windows heterozygous than HAPMIX does (C), leading to its missing some windows of homozygous polar (B) and brown bear (F) ancestry.
